# Plant developmental stage drives the differentiation in ecological role of the maize microbiome

**DOI:** 10.1186/s40168-021-01118-6

**Published:** 2021-08-13

**Authors:** Chao Xiong, Brajesh K. Singh, Ji-Zheng He, Yan-Lai Han, Pei-Pei Li, Li-Hua Wan, Guo-Zhong Meng, Si-Yi Liu, Jun-Tao Wang, Chuan-Fa Wu, An-Hui Ge, Li-Mei Zhang

**Affiliations:** 1 State Key Laboratory of Urban and Regional Ecology, Research Center for Eco-Environmental Sciences, Chinese Academy of Sciences, Beijing, 100085 China; 2 University of Chinese Academy of Sciences, Beijing, 100049 China; 3 Global Centre for Land-Based Innovation, Western Sydney University, Penrith, NSW 2751 Australia; 4 Hawkesbury Institute for the Environment, Western Sydney University, Penrith, NSW 2751 Australia; 5 Faculty of Veterinary and Agricultural Sciences, The University of Melbourne, Parkville, VIC 3010 Australia; 6 College of Resource and Environmental Sciences, Henan Agricultural University, Zhengzhou, 450002 China; 7 Soil and Fertilizer Station of Qilin District, Qujing, Yunnan Province, Qujing, 655000 China

**Keywords:** Crop microbiomes, Temporal dynamics, Soil–plant continuum, Microbiome assembly, Microbial interkingdom networks, Phylloplane microbiome, Metagenomics

## Abstract

**Background:**

Plants live with diverse microbial communities which profoundly affect multiple facets of host performance, but if and how host development impacts the assembly, functions and microbial interactions of crop microbiomes are poorly understood. Here we examined both bacterial and fungal communities across soils, epiphytic and endophytic niches of leaf and root, and plastic leaf of fake plant (representing environment-originating microbes) at three developmental stages of maize at two contrasting sites, and further explored the potential function of phylloplane microbiomes based on metagenomics.

**Results:**

Our results suggested that plant developmental stage had a much stronger influence on the microbial diversity, composition and interkingdom networks in plant compartments than in soils, with the strongest effect in the phylloplane. Phylloplane microbiomes were co-shaped by both plant growth and seasonal environmental factors, with the air (represented by fake plants) as its important source. Further, we found that bacterial communities in plant compartments were more strongly driven by deterministic processes at the early stage but a similar pattern was for fungal communities at the late stage. Moreover, bacterial taxa played a more important role in microbial interkingdom network and crop yield prediction at the early stage, while fungal taxa did so at the late stage. Metagenomic analyses further indicated that phylloplane microbiomes possessed higher functional diversity at the early stage than the late stage, with functional genes related to nutrient provision enriched at the early stage and N assimilation and C degradation enriched at the late stage. Coincidently, more abundant beneficial bacterial taxa like Actinobacteria, *Burkholderiaceae* and *Rhizobiaceae* in plant microbiomes were observed at the early stage, but more saprophytic fungi at the late stage.

**Conclusions:**

Our results suggest that host developmental stage profoundly influences plant microbiome assembly and functions, and the bacterial and fungal microbiomes take a differentiated ecological role at different stages of plant development. This study provides empirical evidence for host exerting strong effect on plant microbiomes by deterministic selection during plant growth and development. These findings have implications for the development of future tools to manipulate microbiome for sustainable increase in primary productivity.

**Video Abstract**

**Supplementary Information:**

The online version contains supplementary material available at 10.1186/s40168-021-01118-6.

## Background

Plants live with large and diverse prokaryotes and eukaryotes (i.e. plant microbiomes) which have coevolved with their hosts and profoundly impact a range of aspects of plant performance [1–4]. For example, some beneficial bacteria and fungi like nitrogen fixer, antagonistic bacteria and mycorrhizal fungi in the rhizosphere and plant compartments can deeply influence plant growth and health via promoting nutrient acquisition, protecting against pathogen attacks, and increasing tolerance to environmental stress [5–8]. Recent studies suggested that plant microbiome assembly and host health are largely influenced by complex and dynamic interactions between the host, microbes, and the environment, but the ecological processes that govern plant-microbiome-environment interactions remain poorly understood [3, 9, 10]. A better understanding of the mechanisms and temporal dynamics of plant microbiome assembly, functions and co-occurrence networks is of significant importance for the development of microbiome-based solutions for sustainable crop production systems [11–14].

Assembly of plant microbiomes starts soon after sowing and develops with plant growth under the influence of deterministic (e.g. selection mediated by biotic and abiotic factors) and stochastic (e.g. random dispersal and drift events) processes [2, 7, 15]. In addition to microbial inheritance and vertical transmission from seed [2, 16, 17], microbes can colonize different plant compartments through dispersal from soil, air and nearby plants, and then form a dynamic community under the integrative effects of host and environmental factors [4, 7, 15, 18, 19]. On the one hand, the plant host has strong selection effects on its microbiomes via host immune system, genetic networks and plant exudates [20–24]. On the other hand, multiple environmental factors such as climate, edaphic properties (e.g. soil pH and nutrients) and human perturbations (e.g. agricultural management regimes) also play important roles in driving plant microbiome assembly [25–29]. It has been reported that plant microbiomes were mainly determined by compartment niche and host species at the plant level, with the phylloplane and rhizoplane acting as an important interface between the host and the environment [30–34]. Some recent studies also highlighted the significant contribution of plant developmental stages on plant microbiome assembly [35–37]. This is expected given that plant physiological requirement and composition of plant exudates vary with its growth [9, 24, 38, 39]. Meanwhile, the effects of plant developmental stages on microbiome also include the effects from season-dependent environmental factors like air, dust, rainfall and temperature. However, we still lack a comprehensive understanding on the mechanisms of microbiome assembly along with plant developmental stage in field, particularly across the soil–plant continuum in which microbiomes are interactively influenced by multiple host and environmental factors like climate, edaphic factors and fertilization regimes.

In addition to host and environmental factors, the assembly and stability of the plant microbiome were also strongly affected by microbe-microbe interactions [2, 40, 41]. Potential microbial interactions across different habitats can be characterized using microbial co-occurrence network analysis [41–43]. The network hubs (hub taxa) which frequently interact with other taxa in microbial networks were considered as the mediators and gatekeepers of microbial communities and played a crucial role in plant microbiome development, host nutrient acquirement and fitness [24, 41, 44, 45]. Accordingly, a recent study on *Arabidopsis* root-microbiota showed that microbial interkingdom interactions among bacteria, fungi and oomycetes could greatly promote *Arabidopsis* survival, and that bacteria played a vital role in protecting plants against pathogens and maintaining microbial interkingdom balance for plant health [45]. Knowledge is lacking however on bacterial-fungal interactions along the soil–plant continuum and how these respond to changes across plant developmental stages, and to what extent these complex interkingdom interactions affect the microbiome dynamics and host performance.

In this study, maize grown in two main agricultural production areas with contrasting soil type and climate condition in China and received different fertilization practices was used as a study model. The dynamics of both bacterial and fungal communities were investigated over three developmental stages (seedling, tasseling and mature stages) across 432 samples from soils (bulk soil and rhizosphere), multiple plant compartment niches (rhizoplane, root endosphere, phylloplane, leaf endosphere, and grain), and plastic leaf (representing local environment background). Specifically, our aims were to (1) uncover the mechanisms of soil and crop microbiome assembly along with plant developmental stages during which microbiomes are interactively influenced by plant development and multiple environmental factors such as soil type, climate and fertilization practice; and (2) disentangle the temporal dynamics of microbial interkingdom network and the ecological function of bacterial and fungal communities across plant developmental stages. We hypothesized that (1) plant development would dominate over environmental factors in shaping plant microbiomes and thus exert greater deterministic selection on them, while it might be vice reverse for soil microbiomes, and the phylloplane microbiome would be more dynamic as a result of multiple effect from host selection and seasonal environmental factors; and (2) the microbial composition, interkingdom network patterns and potential function would alter across plant developmental stages as plant physiological status and seasonal environmental factors would vary greatly, and bacteria and fungi may alternatively function at different stages.

## Materials and methods

### Field experiment description and sampling

The field experiments were located in Xuchang, Henan province (XC, 34°08′20.4"N, 113°48′34.9"E; northern China), and Qujing, Yunnan province (QJ, 25°09′40.8"N, 104°01′51.5"E; southwest China). The fertilization trial with maize and wheat/barley rotation was established in spring of 2016 with seven different fertilization treatments as previously described [33, 46]. To estimate the influence of seasonal environmental factors (e.g. air, dust, temperature, rainfall and UV) on maize phylloplane microbiomes, artificial plants made of plastic material with a height of 1.5 m were planted as “background controls” in the field when the maize was sown (Fig. S1).

Sample collections in two sites were performed in June, August and September of 2017, corresponding to maize seedling, tasseling and mature stage, respectively. For each time point, leaf, root, rhizosphere soil, and bulk soil samples were collected from each plot following our previous method [33, 46]. The plastic leaf samples were also collected from each time point and maize grain samples were collected at the mature stage. As the study mainly aimed at the temporal dynamics of soil and crop microbiomes, we focused on six compartments (bulk soil, rhizosphere, rhizoplane, root endosphere, phylloplane and leaf endosphere) in three treatments (control, 80%N, and 80%NS). We also included phylloplane samples from other four treatments (N, 80%NI, 80%NKle, and 80%NSB), as our previous studies suggested that the phylloplane niche was a hotspot of plant-microbe-environment interactions and affected by both host and environments [33]. In total, we collected 432 samples for microbial community analysis. More information on fertilization treatment, field management and sampling is provided in the supplementary materials “Method S1” and our previous publications [33, 46].

All samples for molecular work were transported to the lab on dry ice and stored at −80 °C until further processing. Soil physicochemical characteristics (e.g. pH, NH_4_^+^-N and NO_3_^−^-N, Table S1) and enzyme activities related to C, N, and P cycling (e.g. nitrogenase activity and phosphatase) were measured according to previous protocols [46–48]. The crop yield was measured at each harvest season based on the field harvest [33].

### DNA extraction

The rhizosphere and bulk soil DNA were extracted from 0.4 g soil using the PowerSoil DNA Isolation Kit (MO BIO Laboratories, Carlsbad, CA, USA) according to manufacturer’s instructions. The epiphytic and endophytic microbial cells from the leaves (10–15 g) and roots (3–5 g) were collected following our previous method [33], and subjected to DNA extraction using the PowerSoil DNA Isolation Kit. The epiphytic DNA from the plastic leaves (10–15 g) was obtained using the same method for the maize leaf epiphytic DNA collection. For maize grain, ~ 5 g sample was ground using sterile mortars and pestles with liquid nitrogen, and then DNA was extracted from the 0.4 g resulting powder using the PowerSoil DNA Isolation Kit.

### Amplicon sequencing and bioinformatic analysis

Bacterial 16S rRNA gene V5-V6 region was amplified using primers 799F and 1115R [49], and fungal ITS2 region was amplified using primers fITS7 [50] and ITS4 [51]. Sequencing was performed on the Illumina MiSeq platform with a paired-end protocol. The raw sequences were quality-filtered using USEARCH (v10.0) [52] as previously described [33, 46], and all correct biological reads (i.e. zero-radius operational taxonomic units, ZOTUs) were picked at 100% similarity using unoise3 command [53] with default parameters. Bacterial and fungal sequences were classified using SILVA (v13.2) and UNITE (v8.0) databases, respectively. In total, 11,665,748 bacterial and 23,156,931 fungal high-quality reads from 432 samples were retrieved and sorted into 18,602 bacterial and 9299 fungal ZOTUs (i.e. phylotypes; analogous to amplicon sequence variants). Bacterial functional profiles were predicted using functional annotation of prokaryotic taxa (FAPROTAX) [54]. Fungal functional guilds were inferred (guild assignments with confidence rankings “Highly probable” and “Probable” were retained) using the program FUNGuild [55]. Both bacterial and fungal alpha- and beta-diversity were calculated in QIIME [56]. Bacterial and fungal ZOTU tables were then rarefied to 3130 and 33,000 reads for the alpha-diversity estimates, respectively. For beta-diversity analysis, ZOTU tables were normalized using the cumulative-sum scaling (CSS) method [57]. More information on the amplicon sequencing and bioinformatic analysis are detailed in the supplementary materials “Method S1”.

### Metagenomic sequencing and data mining

To further characterize phylloplane microbiome functions, we selected 9 maize phylloplane (based on the N treatment, 3 replicates × 3 stages) and 9 plastic leaf (3 replicates × 3 stages) DNA samples from the site QJ for metagenomic sequencing using the Illumina NovaSeq platform with a paired-end protocol. Raw sequences were quality-filtered using Trimmomatic (v0.39) [58], and sequences belonging to the maize genome were removed by mapping the data to the maize reference genome (RefSeq assembly accession: GCF_902167145.1) with Bowtie2 (v2.1.0) [59]. Finally, an average of 8.6 Gb of clean data was retrieved for each sample. These high-quality reads were assembled using Megahit (v1.2.9) [60], and then were predicted using Prokka (v1.14.5) [61] and clustered with a 0.95 similarity threshold using CD-HIT (v4.8.1) to generate non-redundant gene catalog. The functional profiles including KEGG Orthology (KO), Carbohydrate-Active Enzyme (CAZyome) and Clusters of Orthologous Groups of proteins (COG) of phylloplane microbiomes were determined using eggNOG databases (v5.0) [62], and rarefied based on the lowest reads among all samples (KO 9850; CAZyome 430; and COG 19,710). The Chao1 index of functional diversity was calculated based on rarefied table in QIIME.

### Statistical analysis

The linear mixed model (LMM) analysis was performed to identify the major drivers of microbial alpha-diversity using the R package “lme4” [63]. The beta-diversity of both bacterial and fungal communities was assessed by computing weighted UniFrac distance matrices and then ordinated using non-metric multi-dimensional scaling (NMDS). The relative contribution of different biotic and abiotic factors on community dissimilarity was tested with PERMANOVA using the Adonis function (R package “vegan”) [64]. To assess the relative importance of determinism and stochasticity in microbiome assembly, we calculated the beta Nearest Taxon Index (βNTI) using null model (999 randomizations) [65] and defined |βNTI|≥ 2 as dominant deterministic processes and |βNTI|< 2 as dominant stochastic processes [66, 67]. Further, deterministic and stochastic processes were partitioned into five ecological processes based on both βNTI and Bray–Curtis-based Raup-Crick Index (RC_Bray_) values, including heterogeneous selection (βNTI <  − 2), homogeneous selection (βNTI >  + 2), dispersal limitation (|βNTI|< 2 and RC_Bray_ > 0.95), homogenizing dispersal (|βNTI|< 2 and RC_Bray_ < – 0.95), and undominated (|βNTI|< 2 and |RC_Bray_|< 0.95) [66, 67].

Microbial interkingdom network analysis at bacterial and fungal genera level was performed using the CoNet [68] in Cytoscape (v3.5) [69] based on Spearman correlation scores (Spearman’s *r* > 0.7 or *r* <  − 0.7; *P* < 0.01). Both bacterial and fungal genera present in at least 10 samples were retained for the network analysis [46]. The networks were visualized in Gephi [70]. The Source Model of Plant Microbiome (SMPM) was estimated using SourceTracker (v1.0) [71]. Differential abundance analysis was performed using EdgeR’s generalized linear model (GLM) approach [72]. Random forest analysis was conducted to identify the most important predictors (with higher value of percentage increase in MSE) for crop yield using the “randomForest” R package [73]. Mantel test was performed to explore Spearman’s correlations between microbial communities, soil physicochemical characteristics, and soil enzyme activities using the “vegan” package [46]. The linear discriminant analysis (LDA) effect size (LEfSe) [74] was applied (Wilcoxon *P*-value < 0.05, logarithmic LDA score > 2) to identify the biomarker functions for the maize phylloplane and plastic leaf microbiomes. All statistical analyses were carried out in R (http://www.r-project.org). Nonparametric statistical test (Kruskal-Wallis test or Wilcoxon test) was performed to evaluate the alpha-diversity difference and the taxonomical difference among different niches and stages. More information on the LMM, PERMANOVA, network analysis, random forest modeling analysis and source tracking analysis are detailed in the supplementary materials “Method S1” and our previous publication [33, 46].

## Results

### Diversity and community assembly of bacterial and fungal microbiomes across three plant developmental stages

**Fig. 1:**
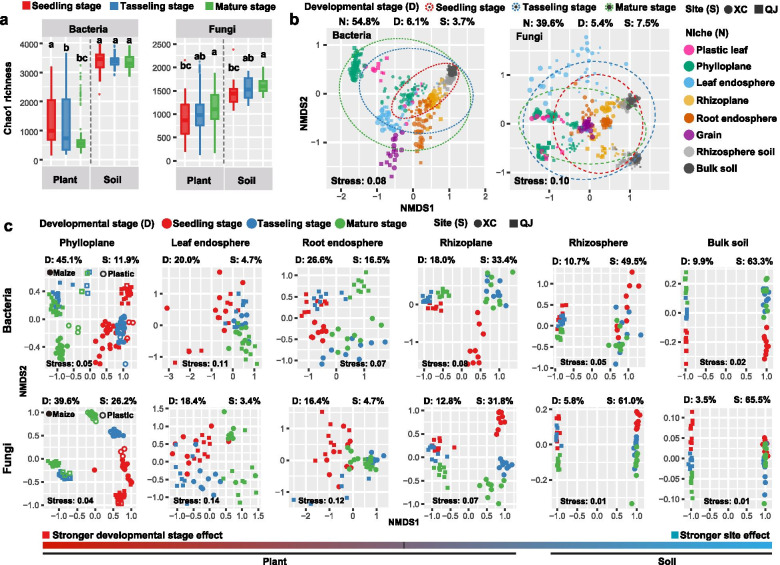
Temporal dynamics of diversity and distribution patterns of crop-associated microbiomes. **a** Alpha-diversity of bacterial and fungal communities in plant compartments (phylloplane, leaf endosphere, rhizoplane and root endosphere) and soils (rhizosphere and bulk soils) across three plant developmental stages. **b** Nonmetric multidimensional scaling (NMDS) ordinations based on weighted UniFrac distance matrices depicting the distribution patterns of bacterial and fungal communities along the soil–plant continuum (*n* = 432). Different letters above the boxes indicate a significant difference determined by nonparametric Kruskal-Wallis test. The relative contribution of different factors on community dissimilarity was tested with PERMANOVA. “N” represents the effect of compartment niche, “D” represents the effect of developmental stage, “S” represents the effect of site. “XC” represents site “Xuchang”, “QJ” represents site “Qujing”. **c** NMDS ordinations based on weighted UniFrac distance matrices of bacterial and fungal communities in each compartment niche (phylloplane: *n* = 144; other niches: *n* = 54)

The linear mixed model analysis suggested that plant developmental stage had a greater influence on both bacterial and fungal Chao1 richness in plant compartment niches (phylloplane, leaf endosphere, rhizoplane and root endosphere) than those in the rhizosphere and bulk soils (Table S2). For plant compartment niches, bacterial Chao1 richness decreased from the seedling stage to the mature stage, while fungi showed opposite pattern (Fig. 1a and Fig. S2a, b).

PERMANOVA analysis and NMDS ordinations indicated that plant developmental stage explained the largest variations (16–45%) in both bacterial and fungal communities in most plant compartment niches (phylloplane, leaf endosphere and root endosphere), with the strongest effect in the phylloplane (bacteria 45.1%, fungi 39.6%) (Fig. 1c; Table S3). In contrast, microbial communities in the rhizoplane, rhizosphere, and bulk soil were primarily explained by site (31–65%) (Fig. 1c; Table S3). Regression analysis based on beta-diversity partitioning further showed that species turnover drove the temporal changes in community composition, and plant developmental stage had the strongest effect on microbial communities in the phylloplane (Fig. S3). At the plant level, microbiome assembly was mainly explained by compartment niche (bacteria 54.8%, fungi 39.6%), followed by developmental stage (bacteria 6.1%, fungi 5.4%) and site (bacteria 3.7%, fungi 7.5%) (Fig. 1b; Table S3). Fertilization practice played a marginal role in driving microbial communities for all samples and samples within each niche (Table S3). Moreover, microbial community dissimilarity among all samples was much lower at the seedling stage than at other stages (Fig. 1b, Fig. S2c). Although maize phylloplane microbiomes significantly differed from plastic leaf microbiomes (*P* < 0.01), both of them showed similar temporal variation patterns (Fig. 1b, c; Table S3).

**Fig. 2:**
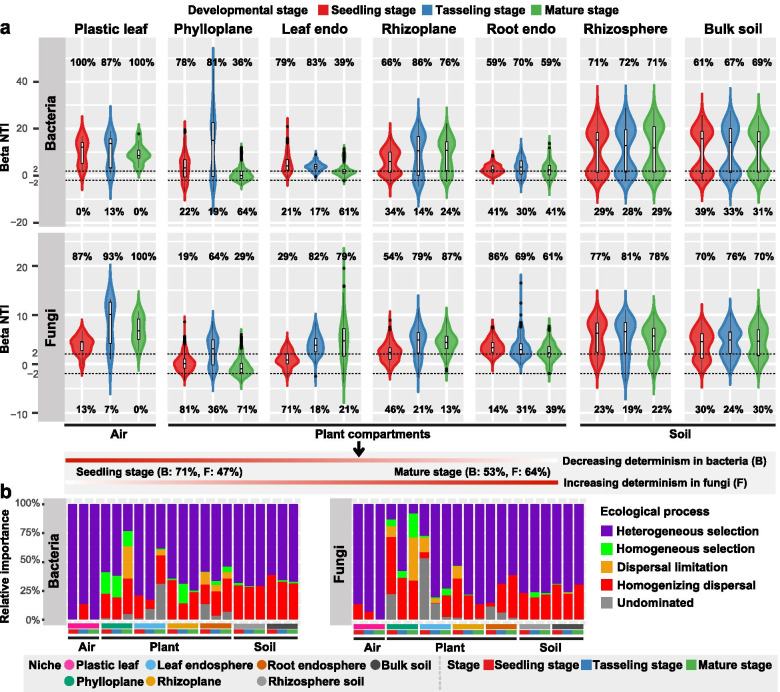
Deterministic and stochastic processes in microbiome assembly. **a** Relative contribution of determinism and stochasticity on microbiome assembly along the soil–plant continuum based on the β-Nearest Taxon Index (βNTI) values. The βNTI values were calculated using null model, and |βNTI|≥ 2 and |βNTI|< 2 represent dominant determinism and stochasticity in driving microbiome assembly, respectively. The percentage above and below the violin plot represent the proportion of the deterministic processes and stochastic processes in microbiome assembly, respectively. “endo” represents “endosphere”. **b** The relative importance of five ecological processes (heterogeneous selection: βNTI <  − 2, homogeneous selection: βNTI >  + 2, dispersal limitation: |βNTI|< 2 and RC_Bray_ > 0.95, homogenizing dispersal: |βNTI|< 2 and RC_Bray_ < – 0.95, and undominated: |βNTI|< 2 and |RC_Bray_|< 0.95) along the soil–plant continuum based on the β-Nearest Taxon Index (βNTI) and Bray–Curtis-based Raup-Crick Index (RC_Bray_)

Null model analysis showed that the relative contribution of deterministic (|βNTI|≥ 2) and stochastic (|βNTI|< 2) processes in crop microbiome assembly were greatly affected by plant developmental stage, particularly for the phylloplane and leaf endosphere (Fig. 2a, b). At the seedling stage, a higher relative contribution of deterministic processes mainly belonging to heterogeneous selection in plant compartments was observed in bacterial communities (~ 71%) than in fungal communities (~ 47%). Conversely, the effects of deterministic processes decreased for bacterial communities (to ~ 53%) but increased for fungal communities (to ~ 64%) at the mature stage (Fig. 2a, b). For the phylloplane and leaf endosphere, a higher relative contribution of stochastic processes mainly belonging to homogenizing dispersal and undominated (e.g. diversification and/or drift) was observed for bacterial community assembly at the late stage and for fungi at the early stage (Fig. 2b). By contrast, both bacterial and fungal communities in the rhizosphere (71–81%), bulk soil (61–76%) and plastic leaf (87–100%) were driven by deterministic processes mainly belonging to heterogeneous selection over the time (Fig. 2a, b). Collectively, deterministic processes exerted a greater influence on crop bacterial community at the early stage and on fungal community at the late stage, respectively.

### Temporal dynamics of microbial interkingdom co-occurrence networks

**Fig. 3:**
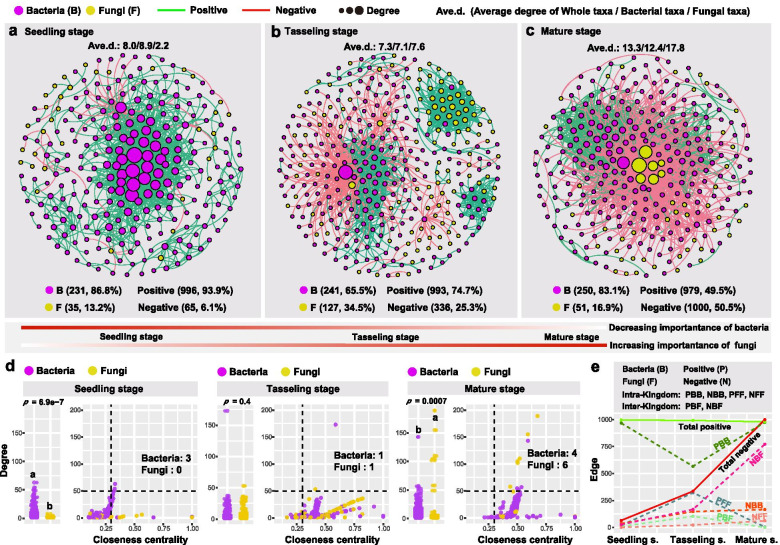
Temporal dynamics of bacterial-fungal interkingdom networks. Co-occurrence network analysis of full dataset (except for maize grain sample, *n* = 414) showing microbial interkingdom network patterns differed among **a** seedling stage, **b** tasseling stage, and **c** mature stage. **d** Comparison of node-level topological features (degree and closeness centrality) between bacterial and fungal taxa at different developmental stages. Different letters indicate a significant difference determined by nonparametric Wilcoxon rank test. **e** Multiple correlations between bacterial and fungal taxa in interkingdom networks at different developmental stages

We further performed co-occurrence network analysis to assess the impact of host developmental stage on bacterial-fungal interkingdom interactions along the plant–soil continuum. Our results showed that microbial interkingdom network patterns shifted clearly across three developmental stages, with differentiated bacterial and fungal roles in the networks during plant development (Fig. 3a–e). Specifically, bacterial taxa had higher network connectivity (i.e. network degree) than fungal taxa at the seedling stage, while the pattern was reversed at the mature stage (Fig. 3a–d). In contrast, fungal network connectivity increased from 2.2 at the seedling stage to 17.8 at the mature stage (Fig. 3a–d). Moreover, the proportion of negative network edges (mainly representing bacteria-fungi interkingdom correlations) markedly increased from 6.1% at the seedling stage to 50.5% at the mature stage (Fig. 3a–c, e). We further defined the “network hubs” as node with high values of degree (> 50) and closeness centrality (> 0.3) in the network, and found 3 network hubs (bacteria 3, fungi 0) at the seedling stage, and 2 (bacteria 1, fungi 1) at the tasseling stage, and 10 (bacteria 4, fungi 6) at the mature stage (Fig. 3d; Table S4). Similar patterns were also recorded in microbial interkingdom functional networks based on function prediction of amplicon sequencing data, with more bacterial functional network hubs (e.g. group “nitrate respiration”) at the first two stages (Fig. S4a–e). In contrast, the highest number of fungal functional network hubs was identified at the mature stage and mainly represented by group “Saprotroph” (Fig. S4a–d).

As for each niche, microbial interkingdom network patterns in plant compartments changed distinctly across three developmental stages, particularly for the phylloplane and leaf endosphere (Fig. S5a–c). The fungal network connectivity and the proportion of fungal nodes significantly increased from the seedling stage to the mature stage in all four plant compartments (Fig. S5a–c). Conversely, the network patterns in the rhizosphere and bulk soil were relatively stable over the three stages (Fig. S5a–c).

### Temporal dynamics of microbiome composition across soil and plant epiphytic and endophytic niches

**Fig. 4:**
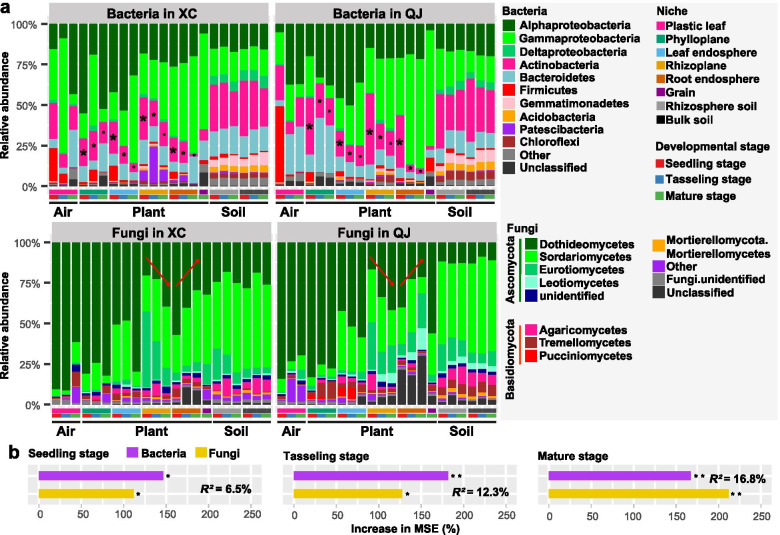
Taxonomic composition and ecological importance of bacterial and fungal microbiomes. **a** Temporal dynamics of both bacterial and fungal community composition at two study sites. “XC” represents site “Xuchang”, “QJ” represents site “Qujing”. Low abundance phyla/classes with less than 1% of the total sequences across all samples are grouped into “Other”. **b** Results from random forest modeling analyses aiming to identify the importance of community composition of bacterial and fungal taxa in predicting crop yield. * *P* < 0.05, ** *P* < 0.001. Increase in the percentage of MSE is equal to the increase in the percentage of the mean square error

Taxonomic classification showed that both bacterial and fungal communities in plant compartment niches varied distinctly across three developmental stages, but not in the rhizosphere and bulk soils (Fig. 4a). The linear mixed model analysis based on the dominant bacterial and fungal phyla/classes (top 10) suggested that plant developmental stage had the strongest effects on bacterial phyla Actinobacteria (*F* = 132.7, *P* < 2.2e–16) and Bacteroidetes (*F* = 78.4, *P* < 2.2e–16), and fungal classes Sordariomycetes (*F* = 34.0, *P* = 8.3e–14) and Dothideomycetes (*F* = 20.0, *P* = 1.6e–8). Notably, we found that Actinobacteria in plant compartments was more abundant at the seedling stage (26.1%) than at the tasseling stage (15.9%) and the mature stage (12.1%, *P* < 0.001) (Fig. 4a, Fig. S6a). In addition, the relative abundance of Dothideomycetes in the rhizoplane increased from the seedling stage to the mature stage (*P* < 0.001), while the Dothideomycetes in the root endosphere showed an opposite pattern (Fig. 4a, Fig. S6b). Notable, Dothideomycetes dominated fungal communities in the plastic leaf and maize phylloplane in both sites and showed no visible variation among three developmental stages (Fig. 4a). Differential abundance analysis at ZOTU level further showed that some members within bacterial families *Burkholderiaceae*, *Microbacteriaceae*, *Streptomycetaceae* and *Rhizobiaceae* were significantly enriched in at least two plant compartments (e.g. phylloplane and rhizoplane) at the seedling stage (Fig. S7a, b; Table S5). Moreover, some members within fungal families *Coniothyriaceae*, *Mycosphaerellaceae*, *Sporidiobolaceae* and *Symmetrosporaceae* were significantly enriched in at least two plant compartments at the mature stage (Fig. S7a, b; Table S5), and some genera within these families were identified as hubs in microbial interkingdom network at the mature stage (Fig. 3a–d; Table S4).

Furthermore, the random forest modeling analysis indicated that bacterial community composition at the seedling and tasseling stages is a strong predictor for crop yield while fungal community composition at the mature stage did so (Fig. 4b). We further correlated distance dissimilarities of bacterial and fungal communities in the rhizosphere and bulk soils with soil nutrients and enzyme activities using Mantel test, and found that NH_4_^+^-N and NO_3_^−^-N were significant correlates of microbial community composition in site XC and QJ, respectively (Fig. S8a–b, Table S6). Notable, soil bacterial communities had significant correlations with soil N cycling-related enzyme activities like nitrogenase and potential nitrification rate (PNR) across three developmental stages in both sites. In contrast, soil fungal communities had significant correlations with soil enzyme activities related to C and P cycling like β-glucosidase and phosphatase (Fig. S8a–b; Table S6).

The Source Model of Plant Microbiome (SMPM) showed that maize-associated bacterial and fungal communities were mainly derived from bulk soils and gradually filtered at different plant compartment niches, and the trends were similar across three developmental stages (Fig. S9a; Table S7). Maize grain potentially selected majority of taxa from leaves (bacteria 46.5%, fungi 28.4%) and roots (bacteria 39.6%, fungi 58.1%) (Fig. S9a). Remarkably, environment-originated (represented by the plastic leaf) microbiomes were important sources of maize phylloplane microbiomes, with an increasing contribution from 52.6 to 87.2% for bacteria and from 86.6 to 92.4% for fungi across three stages (Fig. S9b).

### The functional profiles of phylloplane microbiomes

**Fig. 5:**
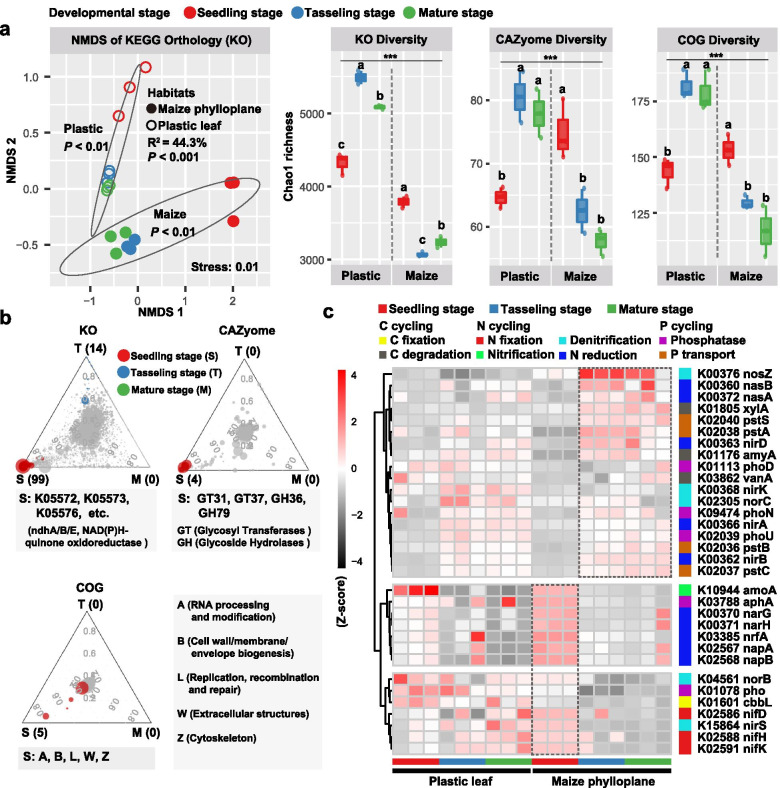
Functional profiles of phylloplane microbiomes. **a** Nonmetric multidimensional scaling (NMDS) ordinations based on Bray–Curtis distance matrices of KEGG Orthology (KO) showing the maize phylloplane microbiome significantly differed from the plastic leaf microbiome (*n* = 18), and the boxplot showing the functional diversity (include KO, CAZyome and COG) of plastic leaf and maize phylloplane microbiomes across three developmental stages. The relative contribution of different factors on microbiome functional composition was tested with PERMANOVA. Different letters above the boxes indicate a significant difference determined by nonparametric Kruskal-Wallis test. **b** Ternary plots depicting functional profiles (include KO, CAZyome and COG) of maize phylloplane microbiome significantly enriched at the seedling stage (FDR, *P* < 0.01). Each circle represents one functional gene/module (i.e. KO, CAZyome and COG), and the size of each circle represents its relative abundance. **c** Heat map exhibiting the relative abundance (Z-score) of functional genes (based on KO) involved in C, N, and P cycling which varied among three developmental stages

Metagenomic analysis indicated that the functional composition (i.e. NMDS ordinations of KEGG Orthology) of maize phylloplane microbiome significantly differed from that of plastic leaf of fake plant (*R*^2^ = 44.3%, *P* < 0.01), and the developmental stage also had significant effect on phylloplane microbiome functions for both maize (*R*^2^ = 50.0%, *P* < 0.01) and fake plant (*R*^2^ = 84.1%, *P* < 0.01) (Fig. 5a). Importantly, the maize phylloplane possessed higher microbiome functional diversity (i.e. Chao1 richness based on KO, CAZyome and COG) at the seedling stage than at other stages, while the plastic leaf showed opposite pattern (Fig. 5a). Differential abundance analysis showed that the number of specifically enriched functional traits (including KO, CAZyome and COG) in maize phylloplane was significantly higher at the seedling stage than at any other stages. Some functional genes/modules involved in quinone oxidoreductase (NAD(P) H, e.g. K05572 and K05573), glycosyl transferases (e.g. GT31) and disease resistance (K13457, probably from host genome) were significantly enriched at the seedling stage (Fig. 5b). Moreover, several C, N and P cycling-related genes showed a varied pattern among three developmental stages (Fig. 5c). Specifically, functional genes involved in nitrate reduction (e.g. *nar*G and *nar*H) in maize phylloplane were more abundant at the seedling stage, while nitrous oxide reductase gene (*nos*Z), N assimilation gene (*nas*A and *nas*B), C degradation- (e.g. *xyl*A and *amy*A) and P transport- (e.g. *pst*A and *pst*B) related genes were more abundant at the other two stages (Fig. 5c). Some functional attributes related to methyl-accepting chemotaxis (K03406) and inorganic ion transport (COG_P) were identified as the biomarker functions for the maize phylloplane while chitin synthase (K00698) and secondary metabolite biosynthesis (COG_Q) were identified for fake plant by LEfSe (Fig. S10a). Although both maize and fake plant shared 39–55% of phylloplane ZOTUs across three stages (Fig. S10b), a stronger depleted effect was observed in the maize phylloplane when compared with fake plant. Some ZOTUs mainly belonging to Alphaproteobacteria and Actinobacteria, like ZOTU7 (*Microbacterium*) and ZOTU9985 (*Sphingomonas*), were significantly enriched in the maize phylloplane over three stages (Fig. S10b).

## Discussion

### Plant developmental stage strongly influences the assembly of plant microbiomes

Uncovering the ecological principles and processes that underpin plant microbiome assembly and developmental dynamics is essential to advance fundamental understanding of coevolution and future application of the crop microbiome to sustainable increase in farm productivity [9–11, 75]. Our results demonstrate that maize microbiome assembly is mainly influenced by compartment niche and developmental stage regardless of farming regions and fertilization regimes. Further, plant microbiomes are more sensitive to plant developmental stage than soil microbiomes in terms of multiple microbial attributes (i.e. alpha-diversity, community structure, assembly processes and interkingdom networks). These findings are consistent with previous studies showing that plant compartment is a determining factor shaping the assembly of plant-associated microbiomes [30–33, 46] and that plant seasonal status has significant effects on microbiomes in grass phyllosphere and *Arabidopsis* rhizosphere [36, 76]. Further, metagenomic analysis in our study revealed that the functional diversity and enriched functional traits in the maize phylloplane varied across three stages. There was a stronger depletion effect in the maize phylloplane in comparison to fake plant phylloplane, and functional gene encoding protein involved in methyl-accepting chemotaxis (K03406, related to signaling in plant–microbe interactions) was significantly enriched in the maize phylloplane. Together, these results indicate that the plant host exerts a strong selection effect to recruit and filter specific microbial taxa and functions from nearby species pool during plant development [21, 77, 78]. Complementary to the previous finding that host selection via plant compartment niche and host genetics plays a dominant role in shaping plant microbiomes assembly [23, 30, 33, 35, 46], this work provides novel evidence that plant developmental stage profoundly influences not only plant microbiome assembly but also their functions.

The effects of plant developmental stage represented the dynamic effects of plant metabolism, exudation and immune-associated traits during plant growth [9, 24, 79], and plant-associated microbes have strong chemotaxis activities towards plant signal molecules such as organic acids and sugars [38, 76, 80–82]. For instance, a recent work revealed that wheat root-released organic carbon varied dramatically across wheat growth stages and correlated with different microbial taxa [38]. It was also suggested that plant exudates and volatiles like coumarins, benzoxazinoids and triterpenes play key roles in shaping plant microbiomes during host growth [20, 77, 81]. On the other hand, the effects of plant developmental stage on microbiome in this study included the effects from season-dependent environmental factors like air, dust and rainwater. By using the artificial plants as “background controls” in the field, the impacts of these environmental factors on plant microbiome assembly were discerned in this study. Our results showed that both maize and fake plant phylloplane microbiomes had similar temporal patterns and shared more than one third of ZOTUs at each stage. Further, environmental source (represented by fake plant phylloplane microbiome) contributed an increasing proportion as the source of the maize phylloplane microbiome over the time. These results presented strong field evidence showing that local air, dust and rainwater are the main sources of crop microbiomes in the phyllosphere. These findings significantly advance our knowledge on the source, driving force and potential function of phyllosphere microbiomes, and further corroborated that the phylloplane acts as an important interface between the host, microbes, and the environment [34, 83–85]. However, we cannot quantify the specific contribution of each environmental factor like dust and rainwater in the current study, and further research is needed to examine this in the future. Our results also showed that plant developmental stage had significant effects on the rhizosphere and bulk soil microbiomes, though it was much weaker than the site effects, implying that plants also have profound influence on soil microbiomes via the rhizosphere effect [5, 39, 79]. Collectively, by examining the temporal dynamics of bacterial and fungal microbiomes in the soil–plant continuum of maize and fake plant phylloplane in geographically distant sites, this study considerably expanded our knowledge on the succession of plant microbiomes and their potential function under different temporal and spatial scales in field.

### The differentiation in ecological roles of bacterial and fungal communities across plant developmental stages

Bacteria and fungi have coevolved with their host for more than 400 million years and greatly contribute to numerous aspects of plant health and productivity [1, 7, 45]. In this study, bacterial-fungal interkingdom interaction patterns distinctly shifted across three developmental stages. Bacterial community possessed higher alpha-diversity and network connectivity at the seedling stage while fungal diversity was higher at the mature stage. Moreover, bacterial and fungal taxa dominated network hubs at the seedling stage and the mature stage, respectively. These suggested that the host may selectively modulate microbial interactions to meet its requirement during plant growth, as microbial network hubs were supposed to play crucial roles in maintaining plant health and nutrient [41, 44]. In addition, bacterial taxa at the first two stages were better predictors of crop yield while fungal taxa at the mature stage did so. This could be explained by the fact that bacterial community may indirectly affect crop productivity by influencing soil enzyme activities and N availability under different fertilization treatments (Fig. S8; Table S1). Similarly, a recent study has suggested that rice root-inhabiting bacterial microbiota can deeply influence nitrogen-use efficiency of the host plants [86]. Metagenomic analysis further corroborated that maize phylloplane microbiome possessed higher functional diversity at the seedling stage than the other two stages. Importantly, more abundant genes associated with nutrient provision and glycosyl transferases were enriched at the seedling stage while N assimilation- and C degradation-related genes were enriched at two late stages.

Based on the limited knowledge on the plant microbiome, it has been proposed that the dynamics of plant microbiome composition are a reflection of the current needs of the host plant [3, 44, 78] and represent the consequence of subtle changes in microbial selection strategy exerted by the host during plant development [1, 24, 78, 87]. Our results therefore supported that bacteria may take a more important ecological role in the plant microbiome and host performance at the early stage, while fungi do so at the late stage. This finding is supported by the null model analysis, which demonstrated the dominant effect of determinism on bacterial community and of stochasticity on fungal community at the seedling stage, but a reverse pattern at the mature stage. In addition, we found that functional gene associated with plant-pathogen interaction (K13457, disease resistance protein RPM1) was significantly enriched at the seedling stage. As the gene is probably derived from the plant genome as the result of biases in plant genome filtering process, the significance of RPM1 enrichment needs further research. The similar bias from shotgun metagenomic sequencing for host microbiomes has also been reported in previous studies [88, 89].

We further found that the negative edges representing bacterial-fungal interkingdom correlations in network increased over the time, implying an increasing competition relationship between bacteria and fungi along plant developmental stages. It was suggested that microbial competitive interaction could positively influence microbiome stability [44, 90, 91]. Our study provided more empirical evidence on this and further supported the argument that the host may facilitate host fitness and plant-microbiome balance by deterministic host selection during plant development. These findings provide new insights into complex interactions among the plant, microbes and the environment and provide essential information for the future development of tools to manipulate crop microbiomes.

### Keystone bacterial and fungal taxa and their ecological functions at different developmental stages

Our results suggested that the composition and potential functions of plant microbiomes change across plant growth, and more abundant Actinobacteria were observed at the seedling stage than at two late stages in plant compartments. Actinobacteria are well known as antagonistic bacteria excreting antibiotic compounds that provide protection against plant pathogens [92–94]. Furthermore, some ZOTUs within families *Burkholderiaceae*, *Streptomycetaceae* and *Rhizobiaceae* were significantly enriched in plant compartment niches at the seedling stage. The members within *Burkholderiaceae* and *Rhizobiaceae* are important diazotrophs and plant growth-promoting rhizobacteria (PGPR) [1, 5, 13], and the members within *Streptomycetaceae* are well-known antibiotic-producing bacteria that are beneficial for plant disease suppression [45, 95, 96]. In addition, bacterial communities in the rhizosphere and bulk soils showed significant correlations with nitrogenase activity across three developmental stages, and the bacterial functional group “nitrite respiration” was identified as the network hubs at the seedling stage. All these suggested that bacterial community takes an ecologically important role in maintaining plant health and nutrient requirement at the early stage.

We further found that the fungal classes Sordariomycetes and Dothideomycetes were more sensitive to plant developmental stage. Previous works have shown that Sordariomycetes and Dothideomycetes are the most dominant fungal taxa in soils and plant compartments, respectively, and that class Dothideomycetes comprises a highly diverse range of fungi including endophytes, epiphytes and plant pathogens [46, 97]. In addition, many members within Dothideomycetes are also identified as saprotrophic fungi functioning in wood and leaf-litter decomposition and nutrient cycling [97, 98]. Notably, fungal communities in both fake plant and maize phylloplanes were predominated by Dothideomycetes in two distant study sites across three developmental stages. It was suggested that Dothideomycetes are the dominant fungal taxa of air microbiomes [98]. This indicated that Dothideomycetes in fake plant and maize phylloplanes might be mainly dispersed from air. Furthermore, some fungal ZOTUs affiliating within families *Coniothyriaceae*, *Mycosphaerellaceae* and *Symmetrosporaceae* were identified as network hubs and significantly enriched in plant compartments at the mature stage. Some members of families *Coniothyriaceae* and *Mycosphaerellaceae* within Dothideomycetes are important saprobes with cellulose- and carbohydrate-degrading ability [98, 99]. Coincidently, we found that most network hubs in both taxonomic and functional networks of the mature stage belonged to fungal functional group “Saprotroph”. Moreover, fungal communities in the rhizosphere and bulk soils had significant correlations with C cycling-related enzymes like β-glucosidase across three developmental stages. These results suggested that fungal taxa play key roles in regulating plant C cycles like decomposition of plant residues at the late stage. This indicates that crop fungal communities may play an increasing ecological role as the decomposers with the aging of the plant, and the host plant may be passively occupied by saprophytic fungi as the consequence of reduced host immunity.

Collectively, our study demonstrates that plant is able to recruit specific microbial taxa with desire functions at different developmental stages. However, the molecular mechanisms governing plant-microbiome interactions during host development and the ecological and biological functions of crop microbiomes in facing climate challenge and achieving sustainable agriculture are not fully understood and need further exploration [11, 100, 101].

## Conclusions

By examining the temporal dynamics of bacterial and fungal communities across soils, multiple plant compartments and fake plant phylloplane at two geographically distant sites, this study provides a systematic understanding on the succession of microbiome composition and their potential functions during plant development. Our results demonstrate that plant developmental stage has a much stronger influence on multiple microbial attributes (i.e. alpha-diversity, community structure, determinism/stochasticity patterns and interkingdom networks) in plant compartment niches than in soils, with the strongest effect in the phylloplane. We further found that air is an important source of phylloplane microbiomes, which were strongly co-shaped by plant growth and seasonal environmental factors. Furthermore, we demonstrated that the ecological role of bacterial and fungal community significantly shifts with plant development, along which bacteria take a more important role in maintaining plant health and nutrient requirement at the early stage while fungi take an increasing role in regulating plant C cycles at the late stage. Additionally, we found a dominant effect of determinism on bacterial communities at the early stage and on fungal communities at the late stage in plant compartments. Together these results suggest that the host has a strong selective modulation effect on the composition and potential functions of plant microbiomes during plant development. These findings significantly advance our fundamental understanding of plant-microbiome interactions and provide critical new knowledge for future synthetic community research and the development of microbiome tools to enhance plant protection and agriculture production in a sustainable way.

## Supplementary Information

**Additional file 1. ** Supplementary information.

## Data Availability

All raw sequencing data have been submitted to the NCBI Sequence Read Archive (SRA) database under the accession numbers PRJNA679910 (16S), PRJNA679909 (ITS), and PRJNA679917 (metagenomics).
