# Supplementary material for: Plant developmental stage drives the differentiation in ecological role of the maize microbiome
Source: Microbiome. 2021 Aug 13;9:171. doi: 10.1186/s40168-021-01118-6 (PMC8364065; doi:10.1186/s40168-021-01118-6)
Supplement: Supplementary file 2 — Additional file 1. Supplementary information. [file 40168_2021_1118_MOESM2_ESM.docx]

## Supporting Information

**Plant** **developmental stage drives** **the differentiation in ecological role of the maize microbiome**

Chao Xiong^1,2^, Brajesh K. Singh^3,4^, Ji-Zheng He^1,5^_,_ Yan-Lai Han^6^, Pei-Pei Li^6^, Li-Hua Wan^7^, Guo-Zhong Meng^7^, Si-Yi Liu^1,2^, Jun-Tao Wang^1,2^, Chuan-Fa Wu^1,6^, An-Hui Ge^1,2^, Li-Mei Zhang^1,2*^

^1^State Key Laboratory of Urban and Regional Ecology, Research Center for Eco-Environmental Sciences, Chinese Academy of Sciences, Beijing 100085, China

^2^University of Chinese Academy of Sciences, Beijing 100049, China

^3^Global Centre for Land-Based Innovation, Western Sydney University, Penrith, NSW 2751, Australia

^4^Hawkesbury Institute for the Environment, Western Sydney University, Penrith, NSW 2751, Australia

^5^Faculty of Veterinary and Agricultural Sciences, The University of Melbourne, Parkville, VIC 3010, Australia

^6^College of Resource and Environmental Sciences, Henan Agricultural University, Zhengzhou 450002, China

^7^Soil and Fertilizer Station of Qilin District, Qujing, Yunnan Province, Qujing, 655000, China

*Author for correspondence: Li-Mei Zhang, Email: zhanglm@rcees.ac.cn, Tel: +86-10-6295 3251

**Supplementary methods**

**Method S1** Detailed description of field trial, molecular methods, bioinformatic and statistical analyses.

**Details on field trial and sampling**

The two study sites are about 1800 km far away, and soils at site XC (northern China) and QJ (southwest China) were classified as calcaric cambisols (pH at about 7.5) and chromic cambisols (pH at about 5.0), respectively. The fertilization trials were established in spring of 2016 with seven different fertilization treatments in both study sites: (1) Control (zero nitrogen fertilizer); (2) N (local regime’ N rate at 400 kg N ha^-1^ year^-1^); (3) 80%N (20% N reduction based on N treatment); (4) 80%NS (80%N treatment plus straw covering at a rate of 6000 kg ha^-1^ year^-1^); (5) 80%NI (80%N treatment plus nitrification inhibitor chlorinated pyridine applied at a rate of 1470 g ha^-1^ year^-1^); (6) 80%NKle (80%N treatment plus foliar spraying of an asymbiotic nitrogen-fixing bacteria *Klebsiella variicola* W12 at a rate of 1000 L ha^-1^ year^-1^, 1 × 10^12^ CFU ml^-1^); (7) 80%NSB (80%NS treatment plus biochar addition at a rate of 30,000 kg ha^-1^ every two years). The two field experiments were random block designed with three replicate plots (about 30 m^2^ for each) and managed according to local agriculture practices. The crop varieties planted were the same as those used by local farmers, with *Zea mays* cultivar Zhengdan 958 at site XC and Shidan 8 at site QJ. For all treatments in both sites, P and K fertilizers were applied as basal fertilizers at a rate of 180 kg ha^-1^ P_2_O_5_ and 180 kg ha^-1^ K_2_O for each year, and 60% of N fertilizer (urea) was used as basal fertilizer and the remaining was applied before jointing stage. Fake plants made of plastic material with a height of 1.5 m and leaf size at about were sterilized with 75% ethanol before using, and randomly planted in three buffer strips between any two maize plots as three replicates, and each replicate included 12 individual plastic plants (Fig. S1).

At each plant developmental stage, about 5 individual maize plants were randomly selected from each plot, and 1-2 healthy leaves at mid-upper position of each plant were clipped and immediately placed on ice bag. Rhizosphere soil (defined as those tightly attached to the roots) was collected by shaking the roots, and then the roots of the same plant were clipped and immediately placed on ice bag. The topsoil (0-15 cm) ~20cm away from the roots was collected as bulk soil, with five subsamples thoroughly mixed as a biological sample for each plot. In total, we collected 432 samples at maize seedling, tasseling and mature stages for microbial community analysis, which included 324 samples from six compartments in Control, 80%N, and 80%NS treatments (6 compartments × 3 treatments × 3 replicates × 2 sites × 3 stages), 72 phylloplane samples from remaining four treatments (4 treatments × 3 replicates × 2 sites × 3 stages), 18 plastic leaf samples (3 replicates × 2 sites × 3 stages) and 18 maize grain samples from Control, 80%N, and 80%NS treatments (3 treatments × 3 replicates × 2 sites).

**Details on amplicon sequencing and bioinformatic analysis**

For both bacterial 16S rRNA gene and fungal ITS2 region amplification, each 25μl PCR reaction containing 12.5 μl Premix Taq DNA polymerase (Takara, China), 0.5 μl each primer (10 μM), 2 μl template DNA (~5 ng μl^-1^), and 9.5 μl PCR-grade water. The PCR amplifications (performed in triplicate for each sample) were carried out using the following program for bacterial 16S rRNA gene: 2 min initial denaturation at 94 °C, 30 cycles of 30 s at 94 °C, 30 s at 55 °C, and 45 s at 72 °C, with a final 10-min elongation at 72 °C. The PCR amplifications (performed in triplicate for each sample) were carried out using the following program for fungal ITS2 region: 94 °C for 5 min, 35 cycles of 94 °C for 30 s, 56.5 °C for 30 s, 72 °C for 30 s, and a final elongation at 72 °C for 7 min.

For raw reads quality-filtered analyses, primer sequences and low-quality reads ends with a quality score (Q) below 30 were trimmed. Paired 16S rRNA gene and ITS amplicon reads were merged to a single sequence and the fungal sequences were further trimmed to 200 bp, and then the resultant sequences were quality-filtered (maximum expected error 0.5) in USEARCH. Bacterial ZOTUs assigned to chloroplast, mitochondrial and viridiplantae, and fungal ZOTUs assigned to plant or protist were removed. Both bacterial and fungal ZOTUs represented by less than 2 sequences were removed to avoid possible biases. Bacterial functional profiles were predicted using functional annotation of prokaryotic taxa (FAPROTAX) [[1](#_ENREF_1)], and 25.4% of bacterial ZOTUs were assigned to at least one microbial functional group. Fungal functional guilds were inferred (guild assignments with confidence rankings “Highly probable” and “Probable” were retained) using the program FUNGuild [[2](#_ENREF_2)], and 86.2% of fungal genera were assigned to at least one guild.

**Detail information on statistical analysis**

The effects of multiple factors on microbial alpha diversity was tested with linear-mixed model (LMM) based on Chao1 richness, and significance was assessed using type II ANOVA with Kenward-Rodger approximation of the degrees of freedom. The model formula for linear mixed model (LMM) analysis used at the plant level was: Microbial alpha-diversity ≈ Compartment niche + Developmental stage + Site + Fertilization practice + Compartment niche × Developmental stage + Compartment niche × Site + Compartment niche × Fertilization practice + Developmental stage × Site + Developmental stage × Fertilization practice + Site × Fertilization practice + (1|block). At the plant level, we used a PERMANOVA based on weighted UniFrac distances matrices to identify the main drivers of microbial community structure, with the model formula was: Microbial community dissimilarity ≈ Compartment niche + Developmental stage + Site + Fertilization practice + Compartment niche × Developmental stage + Compartment niche × Site + Compartment niche × Fertilization practice + Developmental stage × Site + Developmental stage × Fertilization practice + Site × Fertilization practice.

**Supplementary figures**


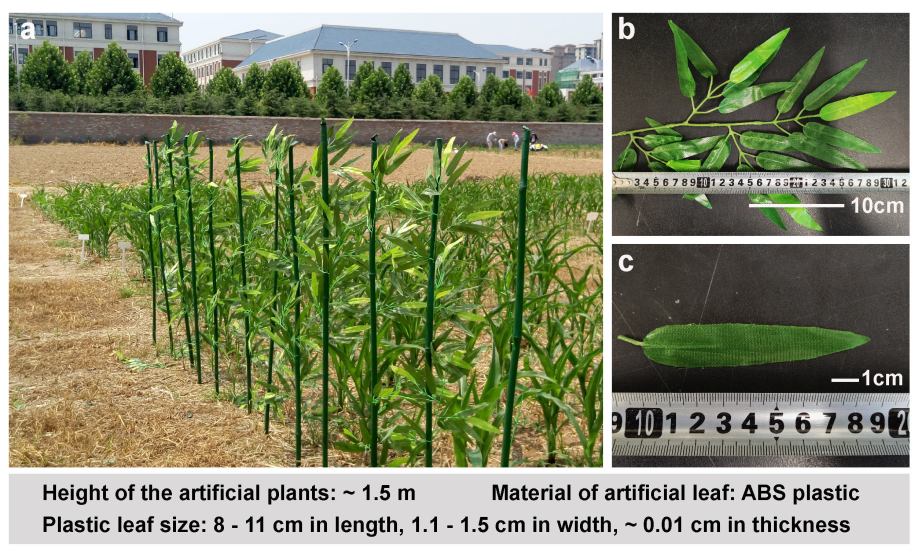


**Fig. S1** The photos and size of artificial plants. a) Plastic plants setup as “background controls” in the field. b) A branch of artificial plant leaves. c) A leaf of artificial plant.


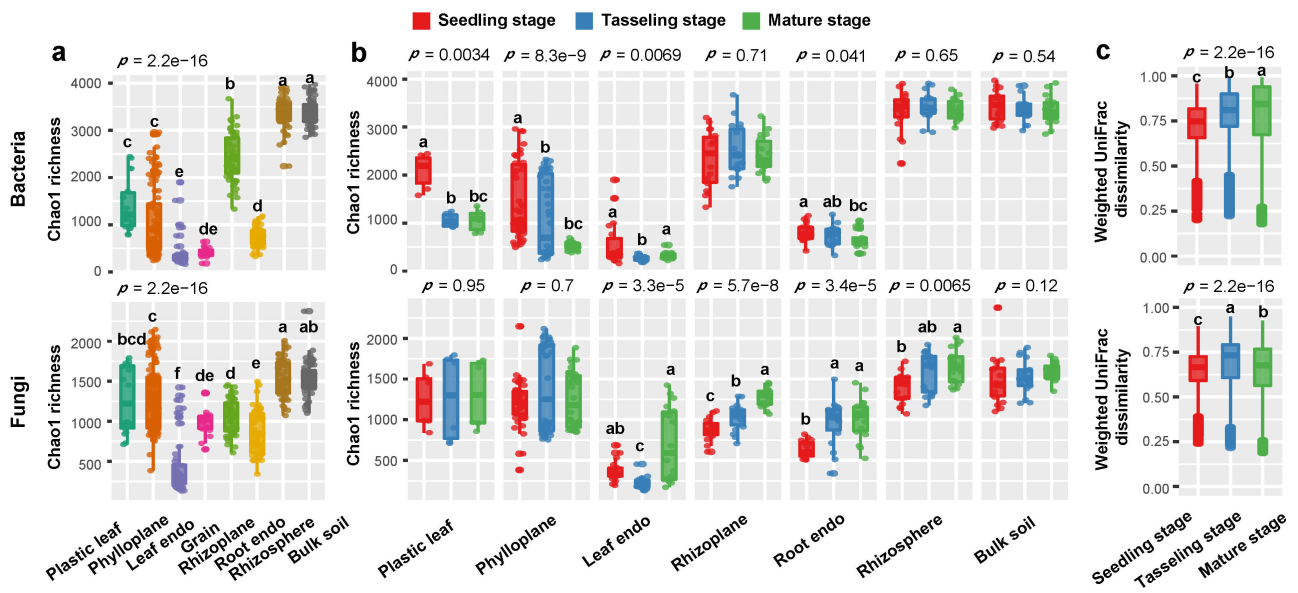
**Fig. S2** Alpha and beta diversity of both bacterial and fungal communities. **a)** Boxplot showing the alpha diversity of both bacterial and fungal communities varied among different compartment niches. **b)** Microbial alpha diversity in each compartment niche varied across three developmental stages. **c)** Microbial community dissimilarity among all samples varied across three developmental stages. Different letters above the boxes indicate a significant difference determined by nonparametric Kruskal Wallis test. “endo” represents endosphere.


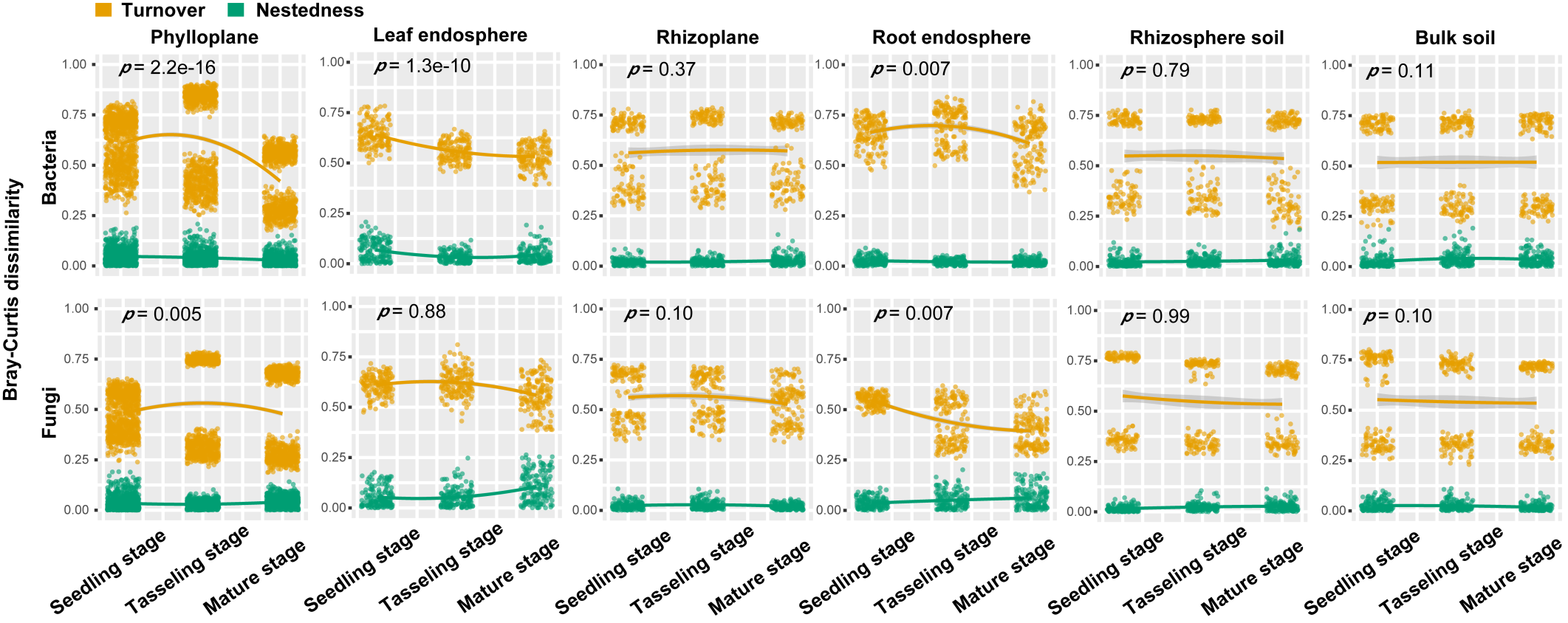


**Fig. S3** Regression analysis based on beta-diversity partitioning of microbial community in each niche. Beta-diversity partitioning indicating that the total beta-diversity of both bacterial and fungal communities in each niche were mainly explained by turnover (i.e. species replacement) rather than by nestedness (i.e. species loss).Temporal regression analysis showing both bacterial and fungal communities in plant compartment niches were more sensitive to plant developmental stage.

**
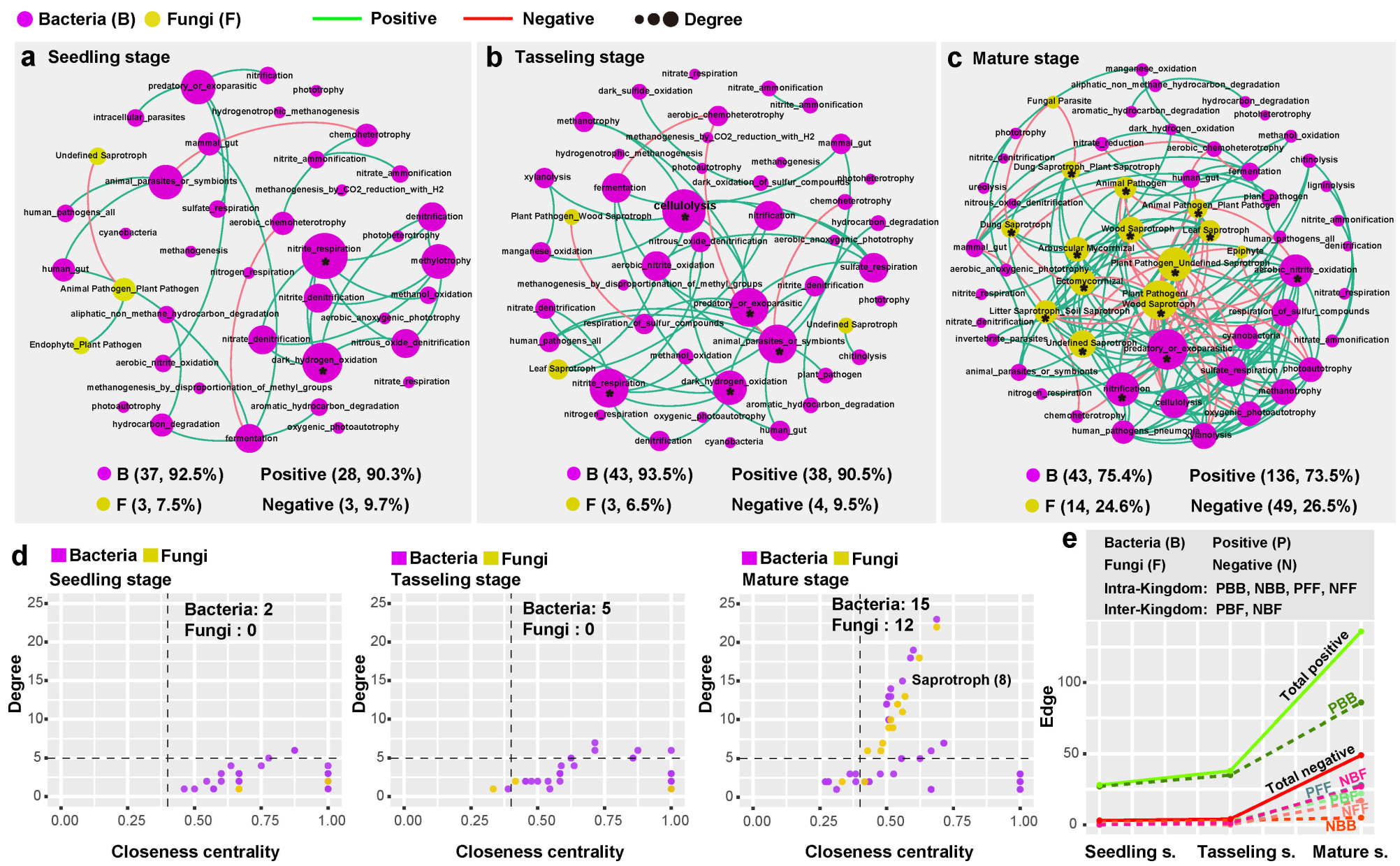
**

**Fig. S4** Dynamics of the microbial interkingdom functional networks. Network analysis based on function prediction of full amplicon dataset (except for grain sample, n = 414) showing microbial interkingdom functional network patterns differed among **a)** seeding stage, **b)** tasseling stage, and **c)** mature stage. **d** The distribution patterns of the “Network hubs” of microbial interkingdom functional networks. The “Network hubs” were defined as node with high values of degree (> 5) and closeness centrality (> 0.3) in the network. **e** Multiple correlations between bacterial and fungal functional groups in interkingdom networks at different developmental stages.


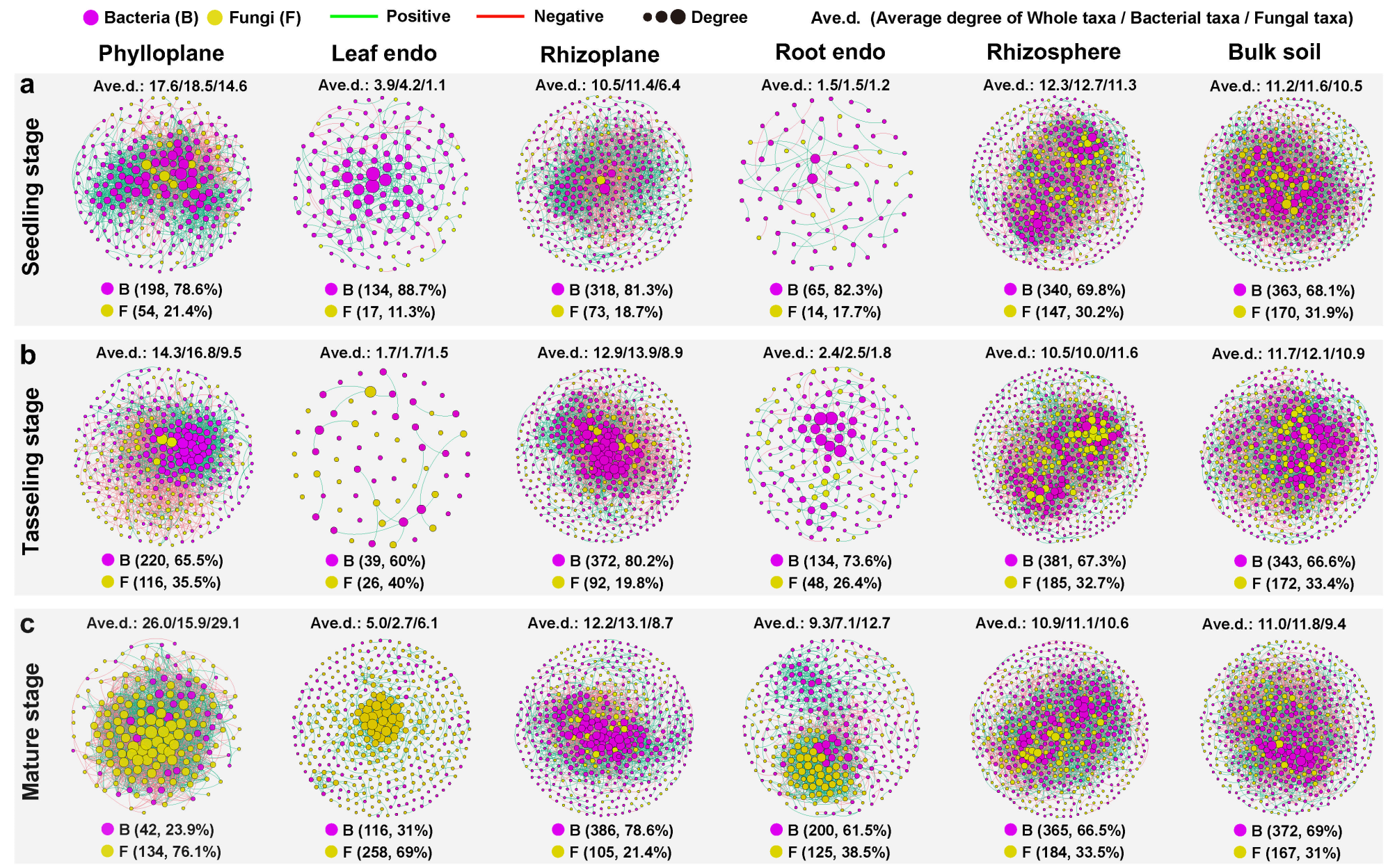


**Fig. S5** Temporal dynamics of microbial interkingdom networks within each niche. Co-occurrence network analysis showing microbial interkingdom network patterns differed clearly among **a)** seeding stage, **b)** tasseling stage, and **c)** mature stage in each plant niche. In contrast, microbiome network patterns in the rhizosphere and bulk soil are relatively stable across three plant developmental stages.


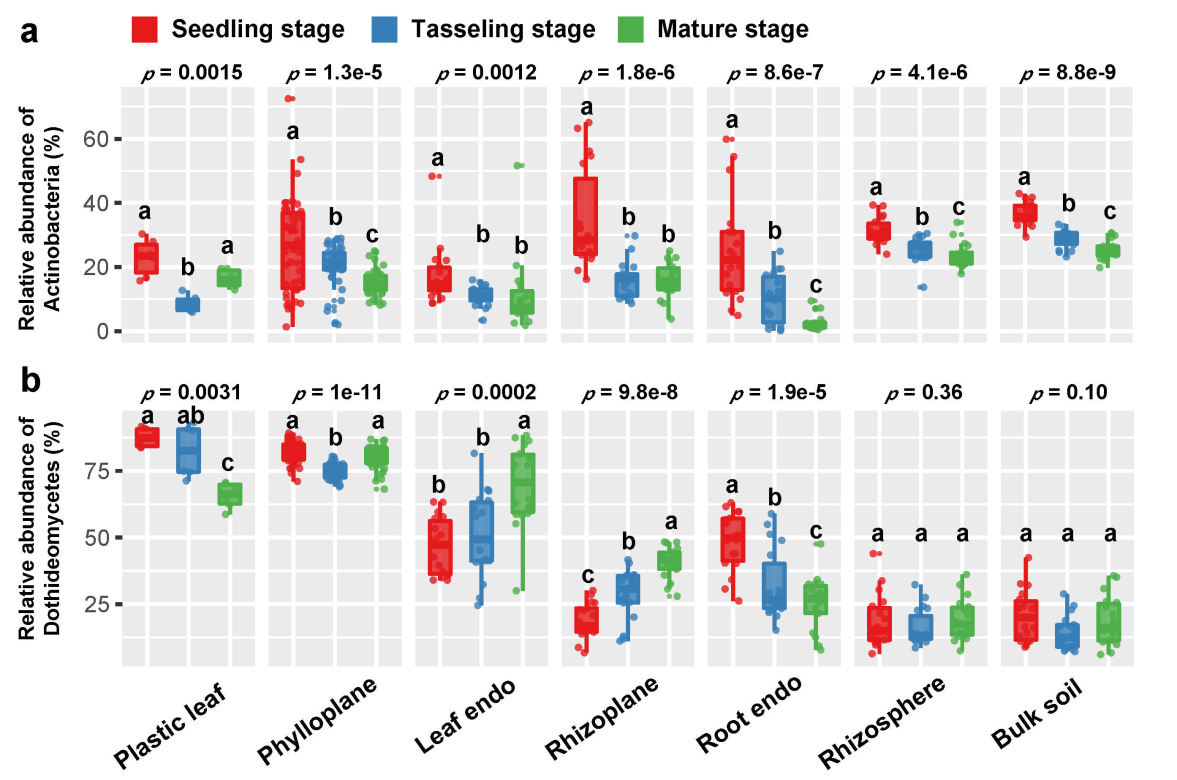


**Fig. S6** Biomarker taxa of microbiome at the phyla/classes level for different developmental stages. Boxplots illustrating the relative abundance of **a)** Actinobacteria and **b)** Dothideomycetes varied among plant developmental stages in different niches. Different letters above the boxes indicate a significant difference determined by nonparametric Kruskal Wallis test.


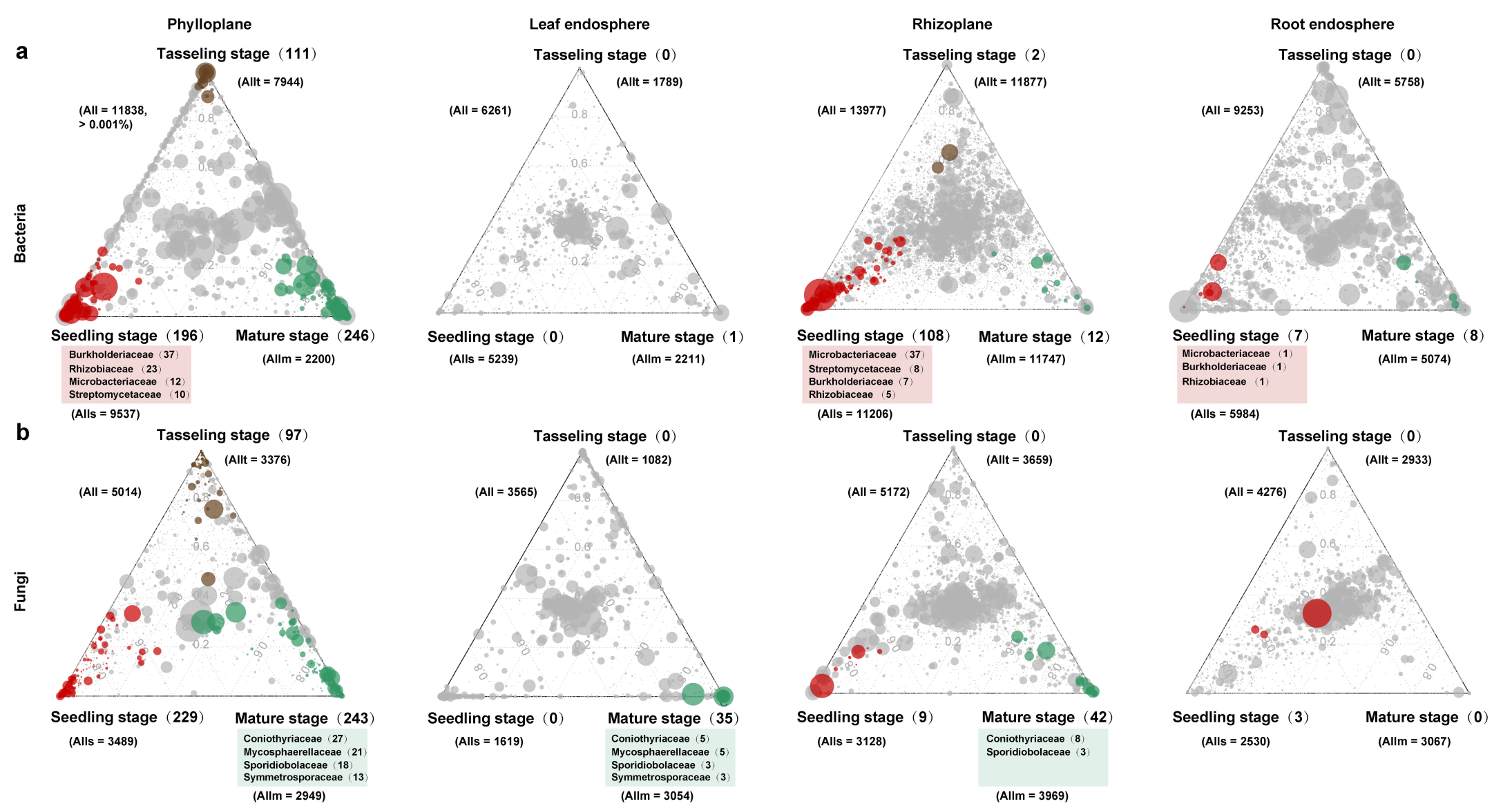


**Fig. S7** Biomarker taxa of crop microbiome at the ZOTU level for different developmental stages. **a-b** Ternary plots depicting bacterial and fungal ZOTUs significantly enriched in three developmental stages within each plant niche (FDR, *p* < 0.01). Each circle represents one ZOTU, and the size of each circle represents its relative abundance. “All” and “Alls/t/m” represent the total number of ZOTUs (RA>0.001%) of all samples and samples in each developmental stage, respectively. The red, brown and green circles represent ZOTUs specifically enriched at seedling, tasseling and mature stage, respectively, whereas gray circles represent ZOTUs that were not significantly enriched at a specific developmental stage. For these significantly enriched ZOTUs, only the taxonomies that shared in different compartments were shown.


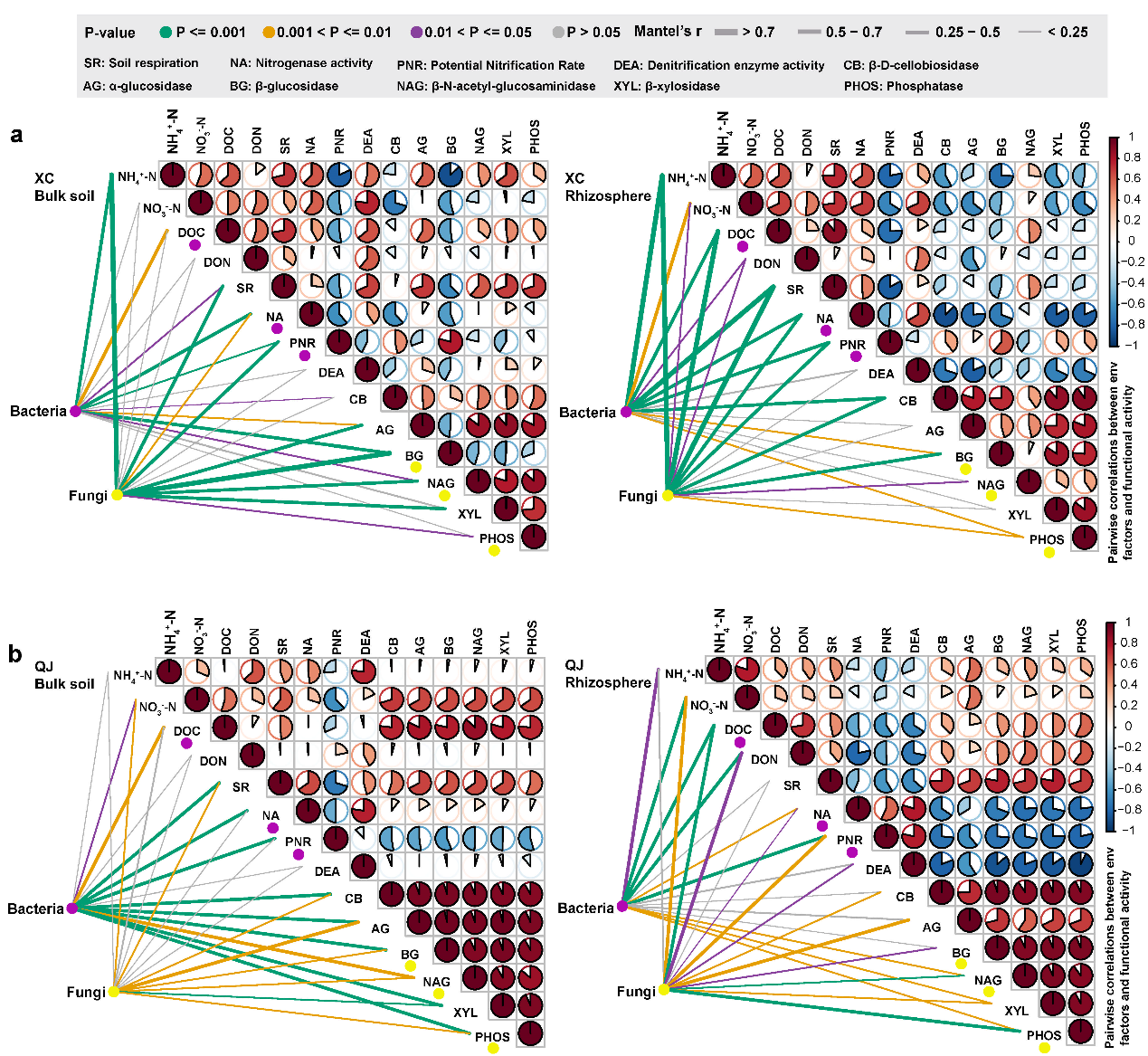


**Fig. S8** Potential drivers and ecological functions of bacterial and fungal communities in the rhizosphere and bulk soils based on Mantel tests. The correlations between microbial communities, soil physicochemical characteristics, and soil enzyme activities in the rhizosphere and bulk soils in site **a)** XC and **b)** QJ. Soil bacterial and fungal communities were related to each environmental factor and enzyme activity by Mantel tests (based on Spearman’s correlations). Edge width corresponds to the Mantel’s r statistic for the corresponding distance correlations (based on Bray-Curtis distances matrices of microbial community and Euclidean distances matrices of environmental factors data), and edge color denotes the statistical significance based on 999 permutations. The proportion of the pie indicates Spearman’s correlation strength, with higher proportion representing higher correlation strength. Factors marked in purple and yellow represent greater correlations with bacterial and fungal communities, respectively.


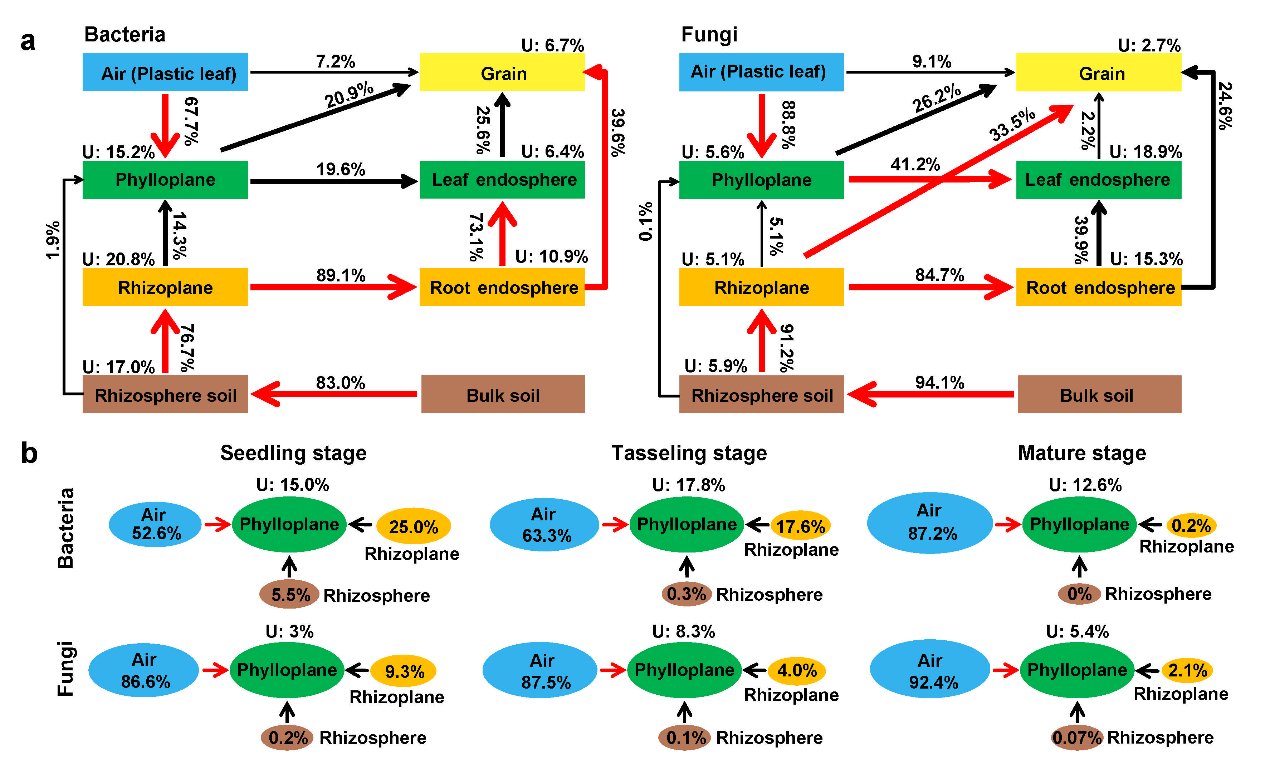


**Fig. S9** Potential sources of crop-associated microbiomes. **a** The Source Model of Plant Microbiome (SMPM) model based on source-tracking analysis showing crop-associated bacterial and fungal communities were derived primarily from bulk soil and were gradually enriched by different plant niches, and **b)** air (represented by the plastic leaf) microbiomes were the important sources of both bacterial and fungal communities in the phylloplane. “U” represents the unknown source, and the thickness of lines equivalent to the source contribution.


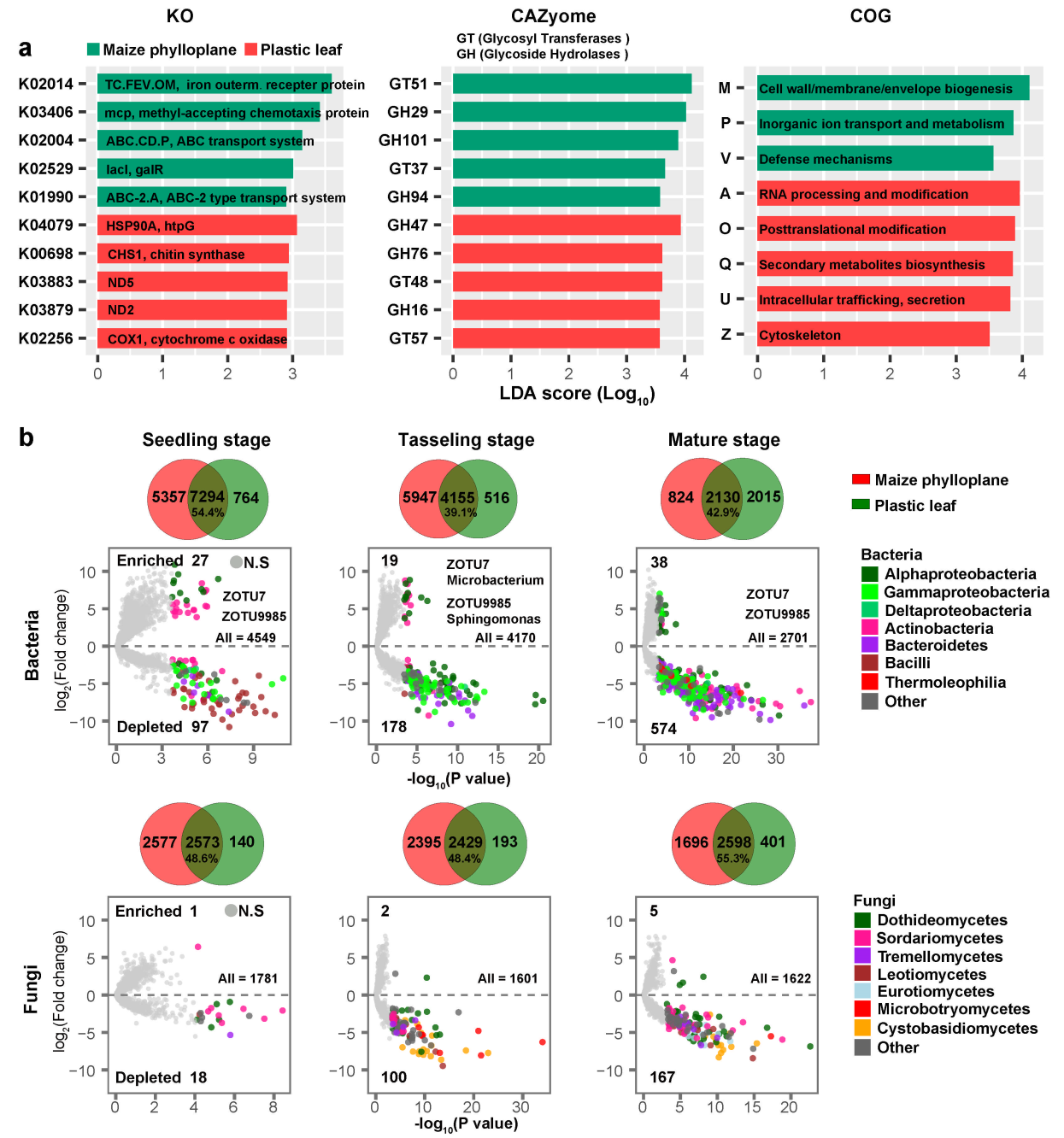


**Fig. S10** Specific microbial taxa and functions between the maize phylloplane and the plastic leaf. **a** LEfSe (linear discriminant analysis effect size) identifying the biomarker functions for the maize phylloplane and plastic leaf microbiomes. Only the top 5 most specific biomarkers were shown. **b** Venn plot showing the shared and specific ZOTUs between the maize phylloplane and the plastic leaf. Volcano plot illustrating the enrichment and depletion patterns of both bacterial and fungal communities in the maize phylloplane compared with the plastic leaf. Each point represents a single ZOTU (RA > 0.1%). Each colored point above the grey line represents an individual enriched ZOTU, and each colored point below the grey line represents an individual depleted ZOTU. The position along the y-axis represents the abundance fold change compared with bulk soil, and x-axis reports the *P* values. “All” represents the numbers of the total ZOTUs (RA > 0.1%) of each developmental stage.

**Supplementary tables**

**Table S1 The soil properties at three developmental stages and crop yields in two study sites**

| Stage | Samples | Treatment | Site XC | | | | | Site QJ | | | | | |
| --- | --- | --- | --- | --- | --- | --- | --- | --- | --- | --- | --- | --- | --- |
|  |  |  | pH | NH_4_^+^-N  (mg kg ^-1^) | NO_3_^-^-N  (mg kg ^-1^) | DOC  (mg kg ^-1^) | DON  (mg kg ^-1^) | pH | | NH_4_^+^-N  (mg kg ^-1^) | NO_3_^-^-N  (mg kg ^-1^) | DOC  (mg kg ^-1^) | DON  (mg kg ^-1^) |
| Seedling stage | Bulk soil | Control | 7.82±0.02a | 12.7±0.5b | 15.9±1.1a | 107.6±15.9a | 25.3±3.3a | 5.45±0.04a | | 9.4±0.9b | 30.1±3.8b | 122.1±14.0a | 32.9±4.6b |
|  |  | 80%N | 7.72±0.01b | 13.0±0.6b | 15.4±1.8a | 97.3±8.0a | 22.2±1.2a | 5.29±0.14a | | 17.9±1.8a | 51.6±3.2a | 121.3±4.2a | 53.0±13.3a |
|  |  | 80%NS | 7.70±0.06b | 16.5±0.4a | 14.0±1.4a | 89.7±11.3a | 22.8±4.1a | 5.33±0.09a | | 14.6±0.8a | 52.3±4.4a | 136.8±3.8a | 45.1±2.1ab |
|  | Rhizosphere | Control | 7.69±0.01a | 17.3±1.4a | 5.1±0.1c | 90.9±9.4a | 17.1±2.1a | 5.40±0.04a | | 11.8±3.1c | 13.1±1.6b | 133.3±14.9a | 29.3±4.6b |
|  |  | 80%N | 7.59±0.04a | 12.3±0.6b | 9.3±0.1a | 98.4±5.3a | 32.1±16.8a | 5.26±0.09a | | 28.4±6.2b | 164.0±14.0a | 142.5±21.0a | 159.2±44.1a |
|  |  | 80%NS | 7.62±0.06a | 10.8±0.2b | 6.6±0.2b | 111.2±19.7a | 26.2±11.3a | 5.26±0.20a | | 63.9±13.2a | 166.3±18.2a | 177.8±37.3a | 207.4±98.4a |
| Tasseling stage^a^ | Bulk soil | Control | 7.89±0.04a | 8.8±0.5b | 13.8±1.1b | 77.3±8.3a | 17.2±0.9b | 5.41±0.1a | | 8.7±1.0a | 11.2±2.7a | 82.7±11.8a | 28.6±1.9b |
|  |  | 80%N | 7.78±0.06a | 8.4±0.2b | 34.9±5.3a | 90.1±9.8a | 43.6±23.1a | 4.99±0.09b | | 15.6±5.8a | 8.7±1.7ab | 86.0±24.5a | 64.0±3a |
|  |  | 80%NS | 7.83±0.01a | 11.4±1.1a | 27.5±1.6a | 86.0±6.1a | 27.1±6.4ab | 5.18±0.04ab | | 17.6±9.6a | 5.9±0.9b | 97.9±10.9a | 43.9±5.8a |
|  | Rhizosphere | Control | 7.83±0.07a | 11.7±0.7a | 3.9±0.5c | 60.1±5.3b | 22.6±0.5b | 5.22±0.09a | | 10.8±1.6a | 30.0±9.8b | 118.0±17.6a | 29.8±2.6a |
|  |  | 80%N | 7.82±0.02a | 10.3±1.0a | 10.1±1.2b | 69.9±5.2ab | 26.6±2.1a | 4.75±0.19b | | 10.2±0.3a | 46.3±4.5a | 124.6±5.7a | 33.3±6a |
|  |  | 80%NS | 7.77±0.02a | 11.7±0.2a | 16.3±2.7a | 73.6±3.7a | 28.2±0.5a | 4.91±0.14ab | | 11.3±0.9a | 59.0±0.5a | 82.3±20.9a | 27.3±5.5a |
| Mature stage | Bulk soil | Control | 7.76±0.03a | 3.3±0.6a | 2.1±0.4a | 79.2±10.7a | 21.4±2.7a | 5.44±0.04a | | 3.9±0.1a | 1.9±0.1b | 118.9±8.2a | 25.6±1.7a |
|  |  | 80%N | 7.68±0.02b | 2.7±0.3a | 2.1±0.2a | 79.7±5.3a | 22.5±1.2a | 5.36±0.02a | | 3.7±0.7a | 11.5±1.5a | 117.0±4.4a | 40.5±2.4a |
|  |  | 80%NS | 7.66±0.03b | 2.7±0.3a | 1.2±0.6a | 71.6±14.7a | 21.4±2.3a | 5.35±0.14a | | 3.3±0.5a | 12.1±4.0a | 120.1±13.4a | 38.5±13.4a |
|  | Rhizosphere | Control | 7.58±0.02a | 2.1±0.5a | 1.8±0.7a | 52.8±2.5b | 18.4±2.5b | 5.46±0.12a | | 7.7±2.8a | 1.4±0.2a | 124.2±21.0a | 38.2±10.3a |
|  |  | 80%N | 7.56±0.03a | 2.8±0.3a | 2.5±1.0a | 60.0±6.7ab | 23.6±1.9a | 5.25±0.09a | | 7.1±1.8a | 1.3±0.6a | 130.3±31.8a | 37.0±7.4a |
|  |  | 80%NS | 7.58±0.01a | 2.8±0.2a | 2.9±0.9a | 65.1±5.0a | 23.9±1.6a | 5.31±0.23a | | 9.5±1.4a | 1.6±0.1a | 128.0±3.9a | 41.9±2.1a |
| Crop yields (kg ha^-1^ season^-1^) | | Treatment | Crop yields in XC site | | | | |  | Crop yields in QJ site | | | | |
|  |  | Control | 7770.6±638.6a | | | | |  | 3891.5±85.7b | | | | |
|  |  | 80%N | 8794.6±385.7a | | | | |  | 7854.3±580.0a | | | | |
|  |  | 80%NS | 10086.0±2297.6a | | | | |  | 8587.9±1004.1a | | | | |

^a^ The data shared with our previous publication [[3](#_ENREF_3)].

The data represents mean ± SD. “XC” represents site “Xuchang, “QJ” represents site “Qujing. Different letters alongside the number indicate a significant difference determined by TukeyHSD test on ANOVA.

**Table S2 Effects of multiple biotic and abiotic factors on microbial alpha diversity**

| Samples | Variables | Bacteria | | | Fungi | | |
| --- | --- | --- | --- | --- | --- | --- | --- |
|  |  | *F* value | df | *P* | *F* value | df | *P* |
| All samples  (n = 432) | Compartment niche | 995.9 | 7 | < 2.2e-16 | 274.4 | 7 | < 2.2e-16 |
|  | Developmental stage | 66.1 | 2 | < 2.2e-16 | 56.3 | 2 | < 2.2e-16 |
|  | Site | 5.9 | 1 | 0.02 | 372.5 | 1 | < 1.2e-13 |
|  | Fertilization practice | 0.7 | 6 | 0.62 | 3.0 | 6 | 0.02 |
| Phylloplane  (n = 126) | Developmental stage | 92.7 | 2 | < 2.2e-16 | 15.0 | 2 | < 6.0e-6 |
|  | Site | 2.0 | 1 | 0.17 | 275.7 | 1 | < 5.0e-16 |
|  | Fertilization practice | 1.7 | 6 | 0.16 | 1.9 | 6 | 0.12 |
| Leaf endopshere  (n = 54) | Developmental stage | 4.4 | 2 | 0.02 | 33.9 | 2 | < 1.0e-7 |
|  | Site | 0.03 | 1 | 0.85 | 12.5 | 1 | 0.004 |
|  | Fertilization practice | 0.4 | 2 | 0.71 | 5.6 | 2 | 0.02 |
| Rhizoplane  (n = 54) | Developmental stage | 1.6 | 2 | 0.21 | 53.3 | 2 | < 1.5e-9 |
|  | Site | 0.1 | 1 | 0.82 | 1.6 | 1 | 0.23 |
|  | Fertilization practice | 1.4 | 2 | 0.27 | 1.3 | 2 | 0.30 |
| Root endosphere  (n = 54) | Developmental stage | 6.0 | 2 | 0.008 | 18.5 | 2 | < 1.4e-5 |
|  | Site | 1.6 | 1 | 0.23 | 0.2 | 1 | 0.69 |
|  | Fertilization practice | 0.1 | 2 | 0.89 | 1.9 | 2 | 0.19 |
| Rhizosphere  (n = 54) | Developmental stage | 1.0 | 2 | 0.36 | 17.5 | 2 | < 2.1e-5 |
|  | Site | 9.4 | 1 | 0.001 | 73.7 | 1 | < 1.8e-6 |
|  | Fertilization practice | 0.1 | 2 | 0.92 | 4.0 | 2 | 0.046 |
| Bulk soil  (n = 54) | Developmental stage | 1.0 | 2 | 0.38 | 2.9 | 2 | 0.08 |
|  | Site | 31.6 | 1 | 0.0001 | 25.1 | 1 | 0.0003 |
|  | Fertilization practice | 1.0 | 2 | 0.40 | 3.6 | 2 | 0.05 |
| Plastic leaf  (n = 18) | Developmental stage | 36.5 | 2 | < 2.2e-16 | 1.3 | 2 | 0.33 |
|  | Site | 0.05 | 1 | 0.54 | 275.3 | 1 | < 7.7e-5 |

The effects of multiple factors on microbial alpha diversity was tested with linear-mixed model (LMM) based on Chao1 richness. Significance was assessed using type II ANOVA with Kenward-Rodger approximation of the degrees of freedom in a linear-mixed model. Relative contributions of interactions between the multiple factors are not shown.

**Table S3** **Effects of** **multiple biotic and abiotic factors on the microbiome assembly**

| Samples | Variables | Bacteria | | Fungi | |
| --- | --- | --- | --- | --- | --- |
|  |  | *R*^2^ | *P* | *R*^2^ | *P* |
| All samples  (n = 432) | Compartment niche | 0.548 | < 0.001 | 0.396 | < 0.001 |
|  | Developmental stage | 0.061 | < 0.001 | 0.054 | < 0.001 |
|  | Site | 0.037 | < 0.001 | 0.075 | < 0.001 |
|  | Fertilization practice | 0.004 | 0.003 | 0.004 | 0.129 |
| Phylloplane  (n = 126) | Developmental stage | 0.451 | < 0.001 | 0.396 | < 0.001 |
|  | Site | 0.119 | < 0.001 | 0.262 | < 0.001 |
|  | Fertilization practice | 0.011 | 0.08 | 0.011 | 0.186 |
| Leaf endopshere  (n = 54) | Developmental stage | 0.200 | < 0.001 | 0.184 | < 0.001 |
|  | Site | 0.047 | 0.003 | 0.034 | 0.02 |
|  | Fertilization practice | 0.022 | 0.879 | 0.035 | 0.104 |
| Rhizoplane  (n = 54) | Developmental stage | 0.180 | < 0.001 | 0.128 | < 0.001 |
|  | Site | 0.334 | < 0.001 | 0.318 | < 0.001 |
|  | Fertilization practice | 0.050 | < 0.001 | 0.032 | 0.039 |
| Root endosphere  (n = 54) | Developmental stage | 0.266 | < 0.001 | 0.164 | < 0.001 |
|  | Site | 0.165 | < 0.001 | 0.047 | < 0.001 |
|  | Fertilization practice | 0.024 | 0.241 | 0.033 | 0.101 |
| Rhizosphere  (n = 54) | Developmental stage | 0.107 | < 0.001 | 0.058 | 0.004 |
|  | Site | 0.495 | < 0.001 | 0.610 | < 0.001 |
|  | Fertilization practice | 0.047 | < 0.001 | 0.019 | 0.124 |
| Bulk soil  (n = 54) | Developmental stage | 0.099 | < 0.001 | 0.035 | 0.035 |
|  | Site | 0.633 | < 0.001 | 0.655 | < 0.001 |
|  | Fertilization practice | 0.020 | 0.046 | 0.019 | 0.156 |
| Plastic leaf  (n = 18) | Developmental stage | 0.534 | < 0.001 | 0.382 | < 0.001 |
|  | Site | 0.148 | < 0.001 | 0.346 | < 0.001 |
| Maize phylloplane VS Plastic leaf  (n = 144) | Habitats | 0.034 | < 0.001 | 0.016 | < 0.001 |

The significance of different factors on microbial community dissimilarity was tested with PERMANOVA (based on weighted UniFrac distances). Relative contributions of interactions between the multiple factors are not shown.

**Table S4 The hubs of microbial interkingdom co-occurrence networks**

| Developmental stage  Count B/F^a^ | Network hubs | Degree | kingdom | Class | Family | Genus | Funguilds |
| --- | --- | --- | --- | --- | --- | --- | --- |
| Seedling stage  3/0 | BZOTU317 | 65 | Bacteria | Thermoleophilia | uncultured | uncultured | na |
|  | BZOTU718 | 54 | Bacteria | Gemmatimonadetes | Gemmatimonadaceae | uncultured | na |
|  | BZOTU495 | 50 | Bacteria | Chloroflexia | Roseiflexaceae | uncultured | na |
| Tasseling stage  1/1 | BZOTU822 | 173 | Bacteria | Alphaproteobacteria | Beijerinckiaceae | Methylobacterium | na |
|  | FZOTU25 | 53 | Fungi | Dothideomycetes | Cladosporiaceae | Cladosporium | Pathotroph/Saprotroph/ Symbiotroph |
| Mature stage  4/6 | FZOTU937 | 189 | Fungi | Tremellomycetes | Bulleribasidiaceae | Dioszegia | Pathotroph/Saprotroph/ Symbiotroph |
|  | FZOTU884 | 155 | Fungi | Cystobasidiomycetes | Symmetrosporaceae | Symmetrospora | Uknown |
|  | BZOTU822 | 143 | Bacteria | Alphaproteobacteria | Beijerinckiaceae | Methylobacterium | na |
|  | FZOTU107 | 110 | Fungi | Dothideomycetes | Coniothyriaceae | Coniothyrium | Pathotroph/Saprotroph |
|  | FZOTU35 | 105 | Fungi | Dothideomycetes | Mycosphaerellaceae | Cercospora | Pathotroph/Saprotroph |
|  | FZOTU25 | 102 | Fungi | Dothideomycetes | Cladosporiaceae | Cladosporium | Pathotroph/Saprotroph/ Symbiotroph |
|  | FZOTU186 | 58 | Fungi | Dothideomycetes | Phaeosphaeriaceae | Neosetophoma | Saprotroph |
|  | BZOTU46 | 57 | Bacteria | Acidobacteria,  Subgroup 5 | uncultured | uncultured | na |
|  | BZOTU982 | 55 | Bacteria | Deltaproteobacteria | Bdellovibrionaceae | uncultured | na |
|  | BZOTU1001 | 52 | Bacteria | Deltaproteobacteria | Myxococcales--BIrii41 | uncultured | na |

^a^ The number of hubs in microbial interkingdom co-occurrence networks, and B/F represents Bacteria/Fungi. na, not applicable.

**Table S5 Biomarker taxa of crop microbiome at the ZOTU level for different developmental stages based on Fig. S6.**

|  | Compartment | Seedling stage | Tasseling stage | Mature stage |
| --- | --- | --- | --- | --- |
| Bacteria | Phylloplane | Burkholderiaceae:37, Rhizobiaceae:23, Microbacteriaceae:12, Streptomycetaceae:10, Azospirillaceae:9 | Corynebacteriaceae:11, Rhodobacteraceae:10, Chitinophagaceae:7, Micrococcaceae:6, Burkholderiaceae:5 | Beijerinckiaceae:95, Sphingomonadaceae:18, Microbacteriaceae:17, Rickettsiaceae:17, Rhizobiaceae:13 |
|  | Leaf endo |  |  | Beijerinckiaceae:1 |
|  | Rhizoplane | Microbacteriaceae:37, Nocardioidaceae:16, Micrococcaceae:8, Streptomycetaceae:8, Burkholderiaceae:7, Rhizobiaceae:5 | Burkholderiaceae:1 | Enterobacteriaceae:4, Burkholderiaceae:2, Chitinophagaceae:2, Beijerinckiaceae:1, Fimbriimonadaceae:1 |
|  | Root endo | Bacillaceae:2, Burkholderiaceae:1, Microbacteriaceae:1, Micrococcaceae:1, Rhizobiaceae:1 |  | Sphingomonadaceae:2, Burkholderiaceae:1, Enterobacteriaceae:1, Flavobacteriaceae:1, Rhizobiaceae:1 |
| Fungi | Phylloplane | Pleosporaceae:22, Chaetomiaceae:15, Teratosphaeriaceae:13, Erysiphaceae:7 | Psathyrellaceae:21, Trichosphaeriaceae:7, Peniophoraceae:5, Lophiostomataceae:3, Melampsoraceae:3 | Coniothyriaceae:27, Mycosphaerellaceae:21, Phaeosphaeriaceae:19, Sporidiobolaceae:18, Symmetrosporaceae:13 |
|  | Leaf endo |  |  | Coniothyriaceae:5, Mycosphaerellaceae:5, Bulleribasidiaceae:4, Sporidiobolaceae:3, Symmetrosporaceae:3 |
|  | Rhizoplane | Nectriaceae:4, Bionectriaceae:1, Ceratobasidiaceae:1, Coniophoraceae:1, Pleosporaceae:1 |  | Coniothyriaceae:8, Bulleribasidiaceae:4, Nectriaceae:3, Sporidiobolaceae:3, Didymellaceae:2 |
|  | Root endo | Didymellaceae:1, Pucciniaceae:1 |  |  |

Microbial taxa colored by purple represents the significant enrichment of bacterial taxa in plant compartments at the seedling stage, and colored by golden yellow represents the significant enrichment of fungal taxa in plant compartments at the mature stage.

**Table S6 Spearman’s correlations** **between microbial communities, soil physicochemical characteristics, and soil enzyme activities based on Mantel tests**

| Variables | | Site XC | | | | | | | | Site QJ | | | | | | | |
| --- | --- | --- | --- | --- | --- | --- | --- | --- | --- | --- | --- | --- | --- | --- | --- | --- | --- |
|  |  | Bacteria of BS | | Bacteria of RS | | Fungi of BS | | Fungi of RS | | Bacteria of BS | | Bacteria of RS | | Fungi of BS | | Fungi of RS | |
|  |  | *r* | *P* | *r* | *P* | *r* | *P* | *r* | *P* | *r* | *P* | *r* | *P* | *r* | *P* | *r* | *P* |
| Environmental  factors | NH_4_^+^-N | **0.43** | **0.001** | **0.55** | **0.001** | **0.52** | **0.001** | **0.54** | **0.001** | -0.01 | 0.501 | 0.32 | 0.013 | 0.03 | 0.309 | 0.16 | 0.108 |
|  | NO_3_^-^-N | 0.11 | 0.126 | 0.33 | 0.002 | 0.06 | 0.225 | 0.21 | 0.014 | **0.21** | **0.025** | **0.45** | **0.001** | **0.28** | **0.004** | **0.30** | **0.002** |
|  | DOC | 0.36 | 0.001 | 0.49 | 0.001 | 0.03 | 0.375 | 0.53 | 0.001 | 0.30 | 0.004 | 0.47 | 0.001 | 0.15 | 0.092 | 0.37 | 0.002 |
|  | DON | -0.09 | 0.747 | 0.23 | 0.018 | -0.17 | 0.946 | 0.21 | 0.018 | -0.02 | 0.530 | 0.45 | 0.002 | -0.01 | 0.506 | 0.33 | 0.009 |
| Functional  activities | SR | 0.27 | 0.009 | 0.56 | 0.001 | 0.47 | 0.001 | 0.56 | 0.001 | 0.32 | 0.004 | 0.08 | 0.156 | 0.26 | 0.017 | 0.13 | 0.064 |
|  | NA | 0.38 | 0.001 | 0.42 | 0.001 | 0.29 | 0.001 | 0.43 | 0.001 | 0.30 | 0.001 | 0.23 | 0.002 | 0.07 | 0.195 | 0.13 | 0.018 |
|  | PNR | 0.28 | 0.001 | 0.45 | 0.001 | 0.31 | 0.001 | 0.50 | 0.001 | 0.34 | 0.001 | 0.32 | 0.001 | 0.05 | 0.237 | 0.30 | 0.003 |
|  | DEA | 0.15 | 0.093 | 0.16 | 0.051 | 0.03 | 0.351 | 0.05 | 0.278 | 0.02 | 0.418 | 0.16 | 0.064 | 0.04 | 0.282 | 0.21 | 0.013 |
|  | CB | 0.12 | 0.034 | 0.42 | 0.001 | 0.00 | 0.496 | 0.33 | 0.001 | 0.38 | 0.001 | 0.13 | 0.079 | 0.23 | 0.010 | 0.24 | 0.010 |
|  | AG | 0.25 | 0.005 | 0.08 | 0.139 | 0.43 | 0.001 | 0.08 | 0.157 | 0.38 | 0.001 | 0.16 | 0.064 | 0.31 | 0.001 | 0.31 | 0.005 |
|  | BG | 0.40 | 0.001 | 0.28 | 0.004 | 0.52 | 0.001 | 0.33 | 0.001 | 0.38 | 0.001 | 0.12 | 0.113 | 0.24 | 0.012 | 0.22 | 0.026 |
|  | NAG | 0.27 | 0.008 | -0.01 | 0.515 | 0.42 | 0.001 | 0.18 | 0.037 | 0.31 | 0.002 | 0.24 | 0.007 | 0.26 | 0.004 | 0.29 | 0.001 |
|  | XYL | 0.16 | 0.044 | 0.11 | 0.118 | 0.40 | 0.001 | 0.12 | 0.081 | 0.36 | 0.001 | 0.22 | 0.009 | 0.29 | 0.001 | 0.29 | 0.002 |
|  | PHOS | 0.14 | 0.079 | 0.27 | 0.002 | 0.21 | 0.012 | 0.22 | 0.004 | 0.36 | 0.001 | 0.26 | 0.005 | 0.25 | 0.009 | 0.36 | 0.001 |

BS (Bulk soil); RS (Rhizosphere soil). The significances was tested based on 999 permutations. DOC, Dissolved organic carbon. DON, Dissolved organic nitrogen. SR, Soil respiration. NA, Nitrogenase activity. PNR, Potential Nitrification Rate. DEA, Denitrification enzyme activity. CB, β-D-cellobiosidase. AG, α-glucosidase. BG, β-glucosidase. NAG, β-N-acetyl-glucosaminidase. XYL, β-xylosidase. PHOS, Phosphatase. Factors coloured by red and blue represents significant correlations with both bacterial and fungal communities of both bulk soil and rhizosphere soil in site XC and QJ, respectively.

**Table S7 The potential sources of crop-associated microbiomes during plant development**

|  | Sink and sources | Air | Phylloplane | Leaf endopshere | Rhizoplane | Root endosphere | Rhizosphere | Bulk soil | Uknown |
| --- | --- | --- | --- | --- | --- | --- | --- | --- | --- |
| Seedling stage | Phylloplane | B: 52.6%, F: 86.6% | na | na | B: 25.0%, F: 9.3% | na | B: 5.5%, F: 0.2% | < 0.01% | B: 15.0%, F: 3% |
|  | Leaf endopshere | na | B: 12.6%, F: 22.9% | na | na | B: 71.0%, F: 55.0% | na | na | B: 15.4%, F: 19.6% |
|  | Rhizoplane | na | na | na | na | na | B: 83.9%, F: 93.6% | na | B: 15.4%, F: 2.6% |
|  | Root endosphere | na | na | na | B: 81.5%, F: 93.1% | na | na | na | B: 17.4%, F: 6.6% |
|  | Rhizosphere | na | na | na | na | na | na | B: 81.4%, F: 96.3% | B: 18.6%, F: 3.7% |
| Tasseling stage | Phylloplane | B: 63.3%, F: 87.5% | na | na | B: 17.6%, F: 4.0% | na | B: 0.3%, F: 0.1% | < 0.01% | B: 17.8%, F: 8.3% |
|  | Leaf endopshere | na | B: 14.1%, F: 33.0% | na | na | B: 82.0%, F: 45.2% | na | na | B: 3.2%, F: 18.5% |
|  | Rhizoplane | na | na | na | na | na | B: 75.2%, F: 89.1% | na | B: 21.0%, F: 5.1% |
|  | Root endosphere | na | na | na | B: 88.8%, F: 79.3% | na | na | na | B: 10.5%, F: 9.7% |
|  | Rhizosphere | na | na | na | na | na | na | B: 83.8%, F: 94.3% | B: 16.2%, F:5.7% |
| Mature stage | Phylloplane | B: 87.2%, F: 92.4% | na | na | B: 0.2%, F: 2.1% | na | B: 0%, F: 0.07% | < 0.01% | B: 15.0%, F: 3% |
|  | Leaf endopshere | na | B: 31.0%, F: 67.7% | na | na | B: 66.3%, F: 19.3% | na | na | B: 1.7%, F: 12.7% |
|  | Rhizoplane | na | na | na | na | na | B: 71.1%, F: 91.0% | na | B: 25.9%, F: 7.7% |
|  | Root endosphere | na | na | na | B: 95.3%, F: 81.6% | na | na | na | B: 4.7%, F: 18.2% |
|  | Rhizosphere | na | na | na | na | na | na | B: 83.8%, F: 91.7% | B: 16.2%, F: 8.3% |

“B” represents bacteria, “F” represents fungi. na, not applicable.

**References**

1. Louca S, Parfrey LW, Doebeli M. Decoupling function and taxonomy in the global ocean microbiome. Science. 2016;353:1272-7.

2. Nguyen NH, Song ZW, Bates ST, Branco S, Tedersoo L, Menke J, et al. FUNGuild: an open annotation tool for parsing fungal community datasets by ecological guild. Fungal Ecology. 2016;20:241-8.

3. Xiong C, Zhu YG, Wang JT, Singh BK, Han LL, Shen JP, et al. Host selection shapes crop microbiome assembly and network complexity. New Phytologist. 2021;229:1091-104.
